# Immunogenomics reveal molecular circuits of diclofenac induced liver injury in mice

**DOI:** 10.18632/oncotarget.7698

**Published:** 2016-02-25

**Authors:** Eun-Hee Lee, Jung-Hwa Oh, Saravanakumar Selvaraj, Se-Myo Park, Mi-Sun Choi, Reinhard Spanel, Seokjoo Yoon, Jürgen Borlak

**Affiliations:** ^1^ Department of Predictive Toxicology, Korea Institute of Toxicology, Daejeon, 305-343, Republic of Korea; ^2^ Department of Human and Environmental Toxicology, School of Engineering, Korea University of Science and Technology, Daejeon, 305-343, Republic of Korea; ^3^ Centre for Pharmacology and Toxicology, Hannover Medical School, 30625 Hannover, Germany; ^4^ Institute for Clinical Pathology, 41747 Viersen, Germany

**Keywords:** diclofenac, NSAID, gene expression profiling, inflammation, immune response

## Abstract

Diclofenac is a non-steroidal anti-inflammatory drug and its use can be associated with severe adverse reactions, notably myocardial infarction, stroke and drug-induced liver injury (DILI). In pursue of immune-mediated DILI mechanisms an immunogenomic study was carried out. Diclofenac treatment of mice at 30 mg/kg for 3 days caused significant serum ALT and AST elevations, hepatomegaly and degenerative changes including hepatic glycogen depletion, hydropic swelling, cholesterolosis and eosinophilic hepatocytes with one animal presenting subsegmental infarction due to portal vein thrombosis. Furthermore, portal/periportal induction of the rate limiting enzyme in ammonia detoxification, i.e. carbamoyl phosphate synthetase 1 was observed. The performed microarray studies informed on > 600 differential expressed genes of which 35, 37 and 50 coded for inflammation, 51, 44 and 61 for immune and 116, 129 and 169 for stress response, respectively after single and repeated dosing for 3 and 14 days. Bioinformatic analysis defined molecular circuits of hepatic inflammation with the growth hormone (Ghr)− and leptin receptor, the protein-tyrosine-phosphatase, selectin and the suppressor-of-cytokine-signaling (Socs) to function as key nodes in gene regulatory networks. Western blotting confirmed induction of fibronectin and M-CSF to hallmark tissue repair and differentiation of monocytes and macrophages. Transcript expression of the macrophage receptor with collagenous structure increased > 7-fold and immunohistochemistry of CD68 evidenced activation of tissue-resident macrophages. Importantly, diclofenac treatment prompted strong expression of phosphorylated Stat3 amongst individual animals and the associated 8- and 4-fold Soc3 and Il-6 induction reinforced Ghr degradation as evidenced by immunoblotting. Moreover, immunohistochemistry confirmed regulation of master regulatory proteins of diclofenac treated mice to suggest complex pro-and anti-inflammatory reactions in immune-mediated hepatic injury. The findings encourage translational research.

## BACKGROUND

Diclofenac is a nonsteroidal anti-inflammatory drug (NSAID) and an antipyretic commonly used in the treatment of inflammatory disorders, rheumatoid arthritis and chronic pain associated with cancer. Repeatedly it was shown that diclofenac use can be associated with drug induced liver injury [1]. The mechanism by which diclofenac causes liver injury remains incompletely understood. However, some clinical and experimental data have been put forward for an improved understanding of hepatotoxicity [2].

Specially, diclofenac is extensively metabolized to 4′-OH and 5′-OH diclofenac by the *Cyp2c9* and *Cyp3a4* monooxygenase system and may also involve increased production of reactive metabolites, most notable diclofenac-2,5- and 1,4-quinone imines. Reactive metabolites can be a leading cause of liver injury, particularly if not sufficiently detoxified via the glutathione redox and conjugation system. Notwithstanding secondary quinone imine metabolites derived from 5-OH and 4′-OH diclofenac glutathione conjugates may be eliminated via biliary excretion especially when bile salt homeostasis is altered. Diclofenac is also metabolized to 4′-OH diclofenac (DCF) acyl glucuronide by the combined activity of CYP2C8 and UGT2B7 in humans and Ugt2b1 in rats. The reactive metabolites are electrophilic and protein-diclofenac adducts have been identified [3]. Additionally, diclofenac acyl glucuronides may accumulate in liver and blood plasma due to saturated drug transport into the biliary canaliculi, and polymorphisms in some ATP-binding cassette transporters (*Mrp2*, *Mrp3* and *Bcrp1*) are associated with cholestatic liver disease as reported for this drug [4].

*In vitro*, diclofenac induced apoptosis through mitochondrial dysfunction and oxidative stress in rat and human hepatocytes and hepatoma cell lines [5] and *Bax/Bak*-mediated mitochondrial outer membrane permeabilization (MOMP) was shown to be a major mechanism of diclofenac-induced hepatotoxicity in a human hepatoma cell line [6, 7]. Moreover, diclofenac inhibited nuclear factor-kappa B (*NF*-κB) activation induced by tumour necrosis factor α (*TNF*α), to result in apoptosis in human and mouse hepatoma cell lines [8]. In rats, a non-hepatotoxic dose of diclofenac co-administered with small amounts of lipopolysaccharide (LPS) caused hepatotoxicity and modest inflammation induced by LPS sensitized hepatocytes to a non-toxic dose of diclofenac [9]. In this regard a T helper 17 cell (Th17)-related immune response was reported in diclofenac-induced acute liver injury in mice [10].

In patient serum samples several protein-adducts of diclofenac and antibodies to diclofenac metabolites were identified. Furthermore, polymorphisms in the gene coding for *Il-10*, *Il-4* and *Il-4r* were associated with diclofenac hepatotoxicity and its outcome [11]. According to clinical research, cholestasis and transient circulating autoantibodies are seen in patients who suffered from acute and chronic hepatitis due to diclofenac [12] and with some patients an autoimmune response was seen after cessation of diclofenac treatment [2, 13], [http://livertox.nlm.nih.gov/Diclofenac.htm]. All these studies suggest an immune mechanism as part of the pathogenesis of diclofenac's idiosyncratic toxicity.

Here we hypothesized for diclofenac a mechanism of hepatotoxicity involving inflammatory reactions. Thus our research focused on an evaluation of acute and repeated treatment responses for up to 14 days. We were particularly interested in identifying molecular circuits of inflammatory response genes and employed whole genome microarrays, qRT-PCR, immunohistochemistry as well as Western blotting to evidence regulation of key molecules in affected livers.

## RESULTS

### Serum markers of liver injury and histopathology findings

**Figure 1:**
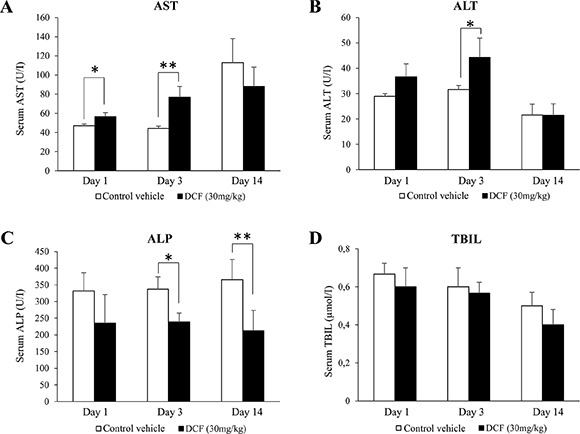
Serum AST, ALT, ALP and TBIL levels in diclofenac-administered mice Diclofenac (30 mg/kg, *i.p*.) was given to mice for day 1, 3 and 14. Result are means ± SD (*n* = 3; *P < 0.05, **P < 0.01).

The serum markers AST, ALT, ALP and total bilirubin (TBIL) were studied after single and repeated treatment, i.e. days 1, 3 and 14. A statistically significant increase in AST and ALT and a reduction in ALP activities (Figure 1) were observed after i.p. administration of diclofenac at 30 mg/kg; however, bilirubin did not differ when control and treatment groups were compared. There was significant variability amongst individual animals to suggest difference in response with some animals being less able to adapt to this treatment regime as was observed after repeated treatment for 14 days (see Figure S1 for individual blood biochemistry data).

To explore dose dependent changes in serum markers of liver injury mice were also treated at 150 mg/kg for up to 3 days. However, a dose dependent change in AST, ALT and ALP activities could not be established as measurements were highly variable amongst individual animals. Moreover, at this dose acute liver failure/death was observed in two out of four animals and additional treatment of two mice caused fatal liver injury in one and acute liver failure in another one. Thus the study was terminated after 48 h of diclofenac treatment.

**Figure 2:**
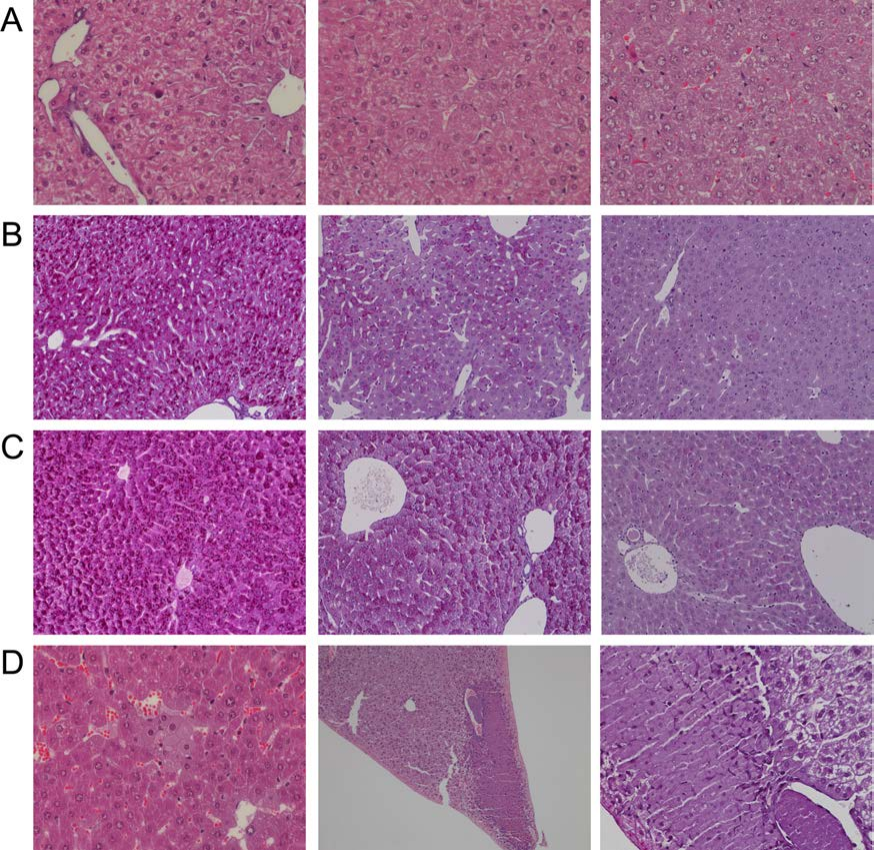
Hematoxylin-eosin and PAS staining of liver sections of Diclofenac treated mice Depicted are representative examples of vehicle control and diclofenac treated animals. (**A**) from the left to the right: H & E stain of vehicle control, single (day 1, 30 mg/kg) and repeated diclofenac treatment of mice (day 3, 150 mg/kg). With control animals the nuclei of hepatocytes appeared quiescent and the cytoplasm of hepatocytes is rich in glycogen (“cloudy”). With treated animals hydropic cytoplasmic swelling of hepatocytes and occasionally eosinophilic hepatocytes are observed (upper left corner, 150 mg/kg animal). The nuclei are activated and the nucleoli are enlarged. The degenerative changes are more pronounced at the higher dose (magnification 200x). (**B**) from the left to the right: PAS staining of vehicle control and single (day 1) diclofenac treatment at 30 and 150 mg/kg. When compared to vehicle controls a significant reduction in the intense PAS staining of hepatocytes was observed to suggest hepatic depletion of glycogen as a result of diclofenac treatment (magnification 100x). (**C**) from the left to the right: PAS staining of vehicle control and after repeated diclofenac treatment (day 3) at 30 and 150 mg/kg. Similar to Panel B and when compared to controls the PAS stain revealed significant depletion of the hepatic glycogen content. Note, the dilatated bile duct in the image given on the right possibly indicates early signs of bile duct obstruction (magnification 100x). (**D**) from the left to the right: H & E stain of repeated diclofenac treatment for 2 days at 150 mg/kg. An example of foamy hepatocytes (“hepatic cholesterolosis”) is given. The second image of panel D (x70 magnification) depicts infarct necrosis observed with an animal after repeated dosing at 30 mg/kg for 3 days. The image on the right is a Diastase-PAS stain and a higher magnification of the infarct necrosis and the fresh thrombosis of the adjacent portal vein (magnification 200x).

Diclofenac treatment caused a significant increase in the liver to body weight ratio (Table 1) and histopathology revealed dose dependent glycogen depletion after single and repeated treatment for up to 3 days (Figure 2). Conversely, hepatic glycogen was only modestly changed after repeated dosing for 14 days (image not shown) to possibly suggest adaptation to diclofenac treatment. Individual animals differed in their responses, nonetheless the Hematoxylin-eosin stain revealed hydropic swelling of heptocytes (Figure 2, Panel A). With some animals repeated diclofenac treatment induced hepatic cholesterolosis (“foamy cytoplasm”) and one animal presented infarct necrosis as a result of portal vein thrombosis (Figure 2, Panel D). Although serum bilirubin laboratory values were within normal range and alkaline phosphatase activity was significantly reduced (Figure 1) the bile ducts in some of the treated animals were dilatated and congested with bile to indicate early signs of bile duct obstruction (Figure 2, panel C, PAS stain illustrating a dilatated and bile fluid filled canaliculi).

**Table 1:** Body and liver weights after diclofenac treatment

Duration	Dosage	Body weight (g)		Liver weight (g)	
		Before administration	After administration	Absolute weight (g)	Relative ratio (%)
Day 1	Vehicle	22.86 ± 1.64	23.63 ± 1.24	1.13 ± 0.03	4.77 ± 0.18 (100)
	30 mg/kg	21.16 ± 1.11	20.90 ± 0.14	1.08 ± 0.03	5.14 ± 0.12_**a**_ (108)
Day 3	Vehicle	20.68 ± 0.86	22.48 ± 0.43_**b**_	1.13 ± 0.03	5.02 ± 0.08 (100)
	30 mg/kg	22.02 ± 1.02	22.84 ± 0.54	1.21 ± 0.03_**a**_	5.31 ± 0.25 (106)
Day 14	Vehicle	24.62 ± 1.03	21.01 ± 1.22_**c**_	0.92 ± 0.13	4.41 ± 0.72 (100)
	30 mg/kg	24.80 ± 0.83	20.43 ± 0.84_**c**_	1.05 ± 0.06	5.14 ± 0.18 (117)

Values represent the means ± standard deviation (SD). The relative organ weight was calculated using the ratio of liver weight to body weight and is represented as a percentage of the total body weight.

*P*-values were calculated using the Student's *t*-test and significance is defined by

a *P* < 0.05; *vs*. corresponding control vehicle.

b *P* < 0.05; *vs*. corresponding before administration;

c *P* < 0.01; *vs.* corresponding before administration.

**Figure 3:**
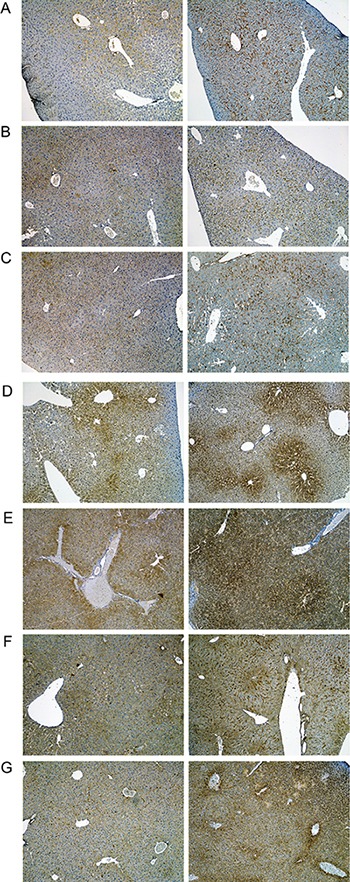
Immunohistochemistry of CD68 in livers of control and diclofenac treated mice (**A**) from the left to the right: Control and single dose 30 mg/kg diclofenac (day 1). (**B**) from the left to the right: Control and repeated dose 30 mg/kg diclofenac (day 3). (**C**) from the left to the right: Control and single dose 150 mg/kg diclofenac (day 1). Diclofenac induced a predominant sinusoidal expression of the CD68 antigen. We also investigated the morphology of sinusoidal endothelial cell (SEC) using the CD31 marker and found SECs to be intact (Images are not shown). **Immunohistochemistry of the macrophage colony stimulating factor (mCSF) in livers of control and diclofenac treated mice.** (**D**) from the left to the right: Control and single dose 30 mg/kg diclofenac (day 1). (**E**) from the left to the right: Control and repeated dose 30 mg/kg diclofenac (day 3). (**F**) from the left to the right: Control and repeated dose 30 mg/kg diclofenac (day 14). (**G**) from the left to the right: Control and repeated dose 150 mg/kg diclofenac (day 3). Magnifications 40x.

**Figure 4:**
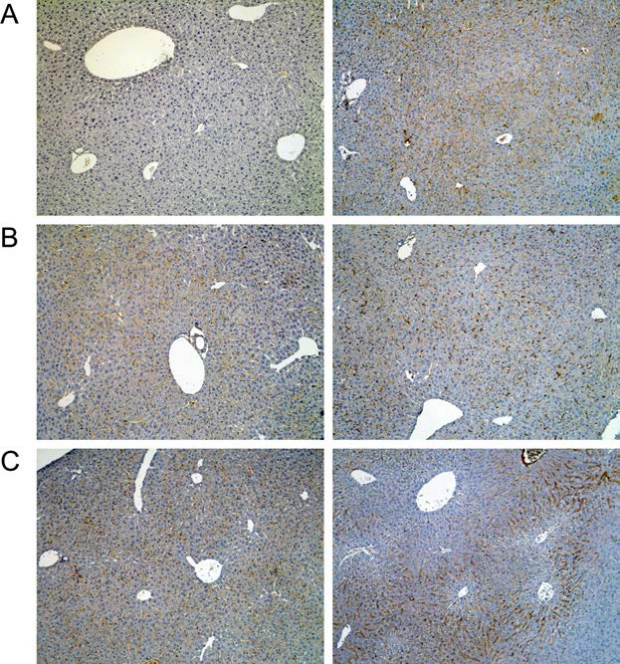
Immunohistochemistry of the lipopolysaccharide binding protein in livers of control and diclofenac treated mice (**A**) from the left to the right: Control and single dose 30 mg/kg diclofenac (day 1). (**B**) from the left to the right: Control and repeated dose 30 mg/kg diclofenac (day 3). (**C**) from the left to the right: Control and single dose 150 mg/kg diclofenac (day 1). Magnifications 60x.

**Figure 5:**
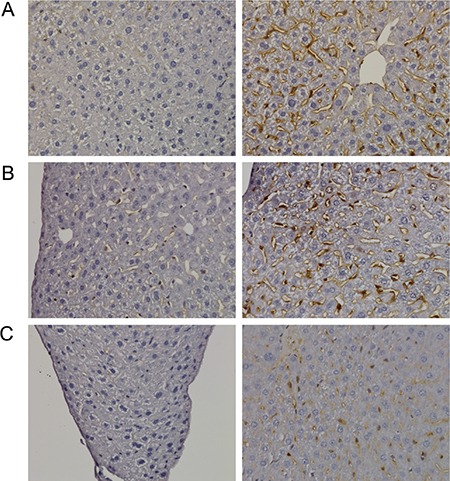
Immunohistochemistry of Ki67 in livers of control and diclofenac treated mice (**A**) from the left to the right: Control and single dose 30 mg/kg diclofenac (day 1). (**B**) from the left to the right: Control and repeated dose 30 mg/kg diclofenac (day 3). (**C**) from the left to the right: Control and single dose 150 mg/kg diclofenac (day 1). Magnifications 240x.

**Figure 6:**
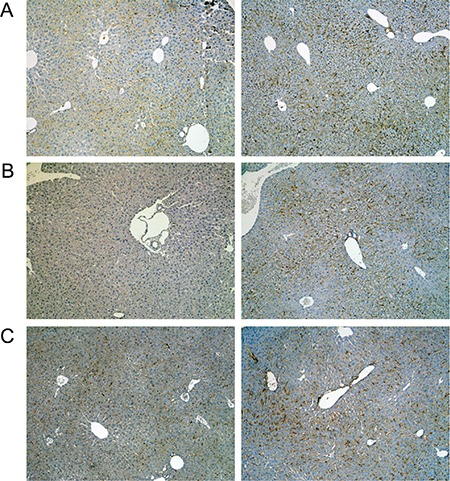
Immunohistochemistry of the T-cell protein tyrosine phosphatase in livers of control and diclofenac treated mice (**A**) from the left to the right: Control and single dose 30 mg/kg diclofenac (day 1). (**B**) from the left to the right: Control and repeated dose 30 mg/kg diclofenac (day 3). (**C**) from the left to the right: Control and single dose 150 mg/kg diclofenac (day 1). Magnifications 60x.

A range of immunohistochemistry studies were performed with CD31 evidencing sinusoidal endothelial cells to be intact (not shown), however hepatic sinusoids appeared partially widened. Likewise, the increased staining for CD68 (Figure 3A) marked activated Kupffer cells and appeared to be co-stimulated by the enhanced secretion of the cytokine macrophage colony-stimulating factor (M-CSF) as observed for the centrolobular regions (Figure 3B). Equally, the pronounced sinusoidal and occasionally strong hepatic expression of the acute phase protein lipopolysaccharide binding protein (LBP) reinforces the notion of Kupffer cell activation (Figure 4). Note, LBP is bound by CD14 and transcript expression of this pattern recognition receptor was > 30-fold induced (see Table S9). Further evidence for proliferation of sinusoidal resident antigen-presenting cells was obtained by examining Ki67. An increase in Ki67 positive cells was observed (Figure 5) and it is well established that activated Kupffer cells release cytokines, chemokines, reactive oxygen species and other mediators of inflammation. As the activation of resident macrophages was not confined to regions of harmed hepatocytes diclofenac treatment induced a more general response by augmenting drug induced hepatitis. Alike, the significant regulation of the T-cell protein tyrosine phosphatase (Tc-PTP; Figure 6) is an important finding. Tc-PTP is a key player of the immune system and a negative regulator of Colony-Stimulating Factor 1 (CSF-1) signalling and macrophage differentiation. Note, CSF-1 was significantly repressed in transcript expression by nearly 40% in livers of diclofenac treated mice (see Table S2).

**Figure 7:**
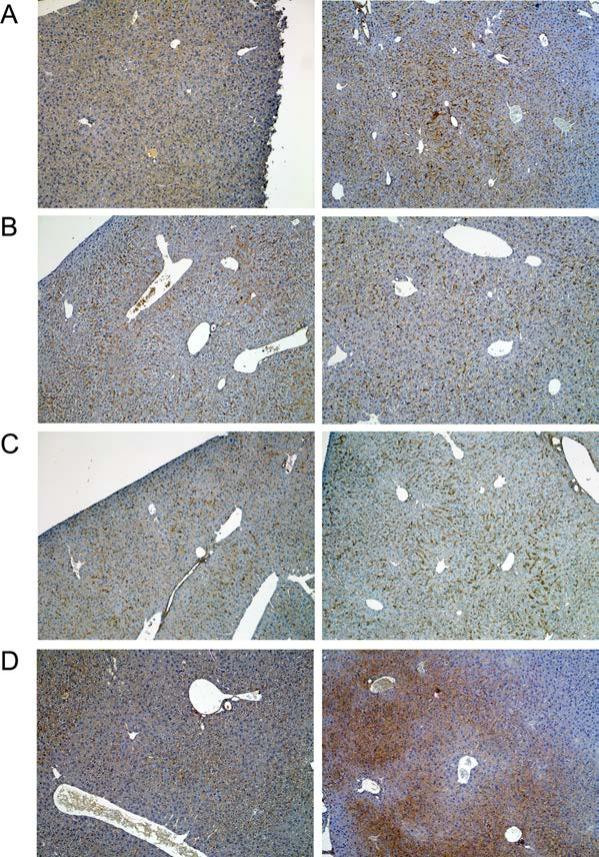
Immunohistochemistry of fibronectin in livers of control and diclofenac treated mice (**A**) from the left to the right: Control and single dose 30 mg/kg diclofenac (day 1). (**B**) from the left to the right: Control and repeated dose 30 mg/kg diclofenac (day 3). (**C**) from the left to the right: Control and repeated dose 30 mg/kg diclofenac (day 14). (**D**) from the left to the right: Control and single dose 150 mg/kg diclofenac (day 1). Magnifications 50x.

**Figure 8:**
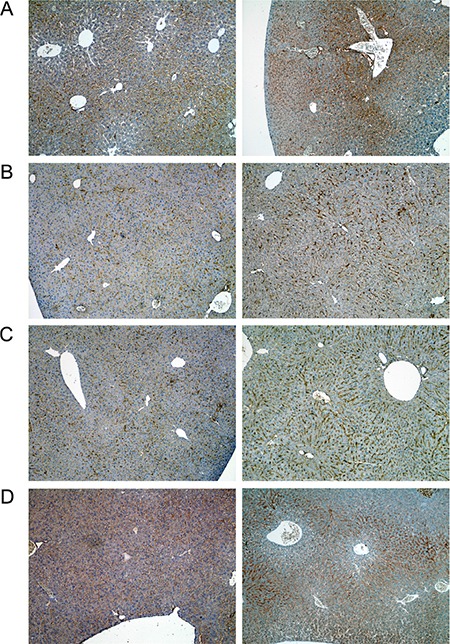
Immunohistochemistry of growth hormone receptor in livers of control and diclofenac treated mice (**A**) from the left to the right: Control and single dose 30 mg/kg diclofenac (day 1). (**B**) from the left to the right: Control and repeated dose 30 mg/kg diclofenac (day 3). (**C**) from the left to the right: Control and repeated dose 30 mg/kg diclofenac (day 14). (**D**) from the left to the right: Control and single dose 150 mg/kg diclofenac (day 1). Magnifications 50x.

**Figure 9:**
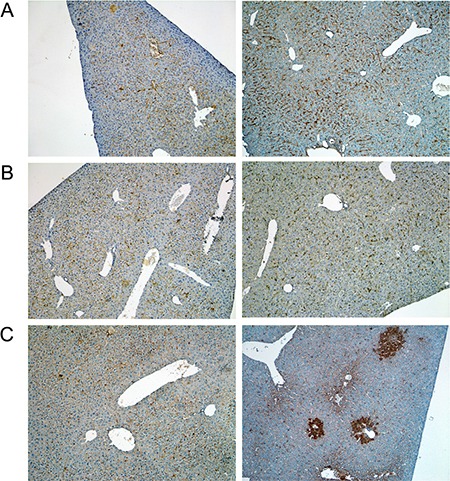
Immunohistochemistry of the leptin receptor in livers of control and diclofenac treated mice (**A**) from the left to the right: Control and single dose 30 mg/kg diclofenac (day 1). (**B**) from the left to the right: Control and repeated dose 30 mg/kg diclofenac (day 3). (**C**) from the left to the right: Control and repeated dose 150 mg/kg diclofenac (day 2). Magnifications 50x.

Moreover, enhanced expression of fibronectin, i.e. a matrix glycoprotein known to influence hepatic survival was strongly upregulated particularly at the 150 mg/kg dose to signify tissue repair (Figure 7, Panel D). Besides, we investigated regulation of the growth hormone receptor (Ghr, Figure 8) and found its expression to be significantly increased in the sinusoids especially of periportal and intermediate regions localised hepatocytes after single treatment (day 1), however Ghr staining was markedly reduced after repeated treatment for 3 days. Importantly, growth hormone signals through Ghr and is known to play a decisive role in liver regeneration. Moreover, diclofenac treatment induced enhanced sinusoidal expression of the leptin receptor (Figure 9); its infrequently increased expression by harmed hepatocytes was also observed. In addition, we investigated the expression of Hep Par 1 (Figure S2) and noted strong cytoplasmic staining of hepatocytes in diclofenac treated animals, however the centrolobular regions were spared. It was demonstrated that Hep Par 1 recognizes the urea cycle rate limiting enzyme carbamoylphosphate synthetase 1. Hence, we evidence ammonia detoxification and observed strong focal staining of hepatocytes surrounding the portal tracts (see day 3, 30 mg/kg). Enhanced expression of the antigen was also observed with disperse hepatocytes as seen at the 150 mg treatment dose (Figure S2, Panel C). Finally, we examined the possibility of liver architectural changes in animals treated repeatedly for 14 days. By employing the Elastica van Gieson and the Gomori silver stain no evidence was obtained for early signs of fibrosis or enhanced deposition of extra cellular matrix into the sinusoids.

### Genomic response of the liver to diclofenac treatment

**Figure 10:**
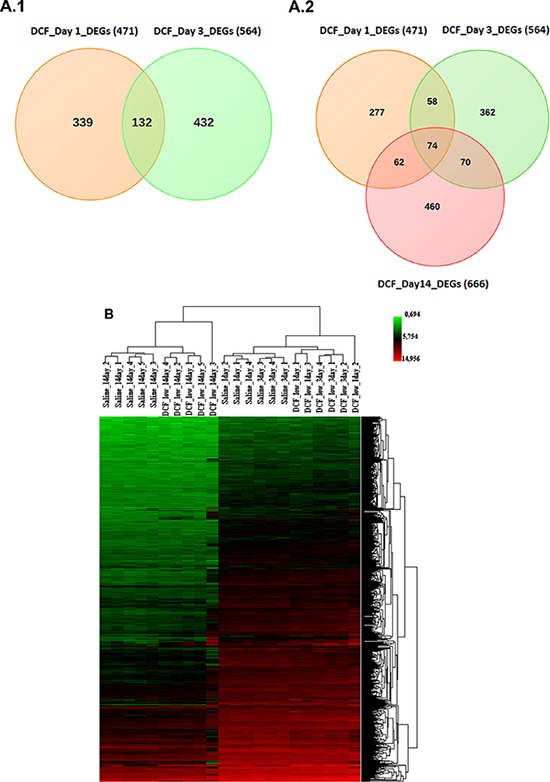
**(A) Differentially expressed genes after single and repeated diclofenac treatment in mice.** Venn diagrams of DEGs induced after single (day 1) and repeated (day 3 and day 14) treatment. A total of 132 genes were found to be regulated in common after single and repeated treatment for 3 day. Furthermore, 74 genes were commonly regulated amongst all treatment groups. (**B**) **Heat map of differentially expressed genes in liver of diclofenac treated mice.** The heat map was generated using average-linkage hierarchical clustering with Euclidean distance. The heat map was generated with the ArrayTrack software version 3.5.0. Depicted is the signal intensity value for differentially expressed genes. The diclofenac day 1, 3 and 14 treatment group were clearly segregated from the vehicle treated control animals.

RNA was extracted from livers of vehicle- and diclofenac (30 mg/kg)-treated mice after single, i.e. 24 h and repeated dosing for 72 h and 14 days. Because of the sudden death of two animals at the 150 mg/kg dose the livers from high dose animals were not used for the microarray experiments. Gene expression profiling of RNA samples was performed with the Affymetrix GeneChip System. Initially, the data were analysed with the GenPlex software version 3.0 [http://genplex.co.kr] and a total of 295 and 467 significantly regulated genes at >1.5-fold change and statistical significance testing (Welch's *t*-test, *p* < 0.05) were determined after single and repeated treatment for 3-days. Additionally, the microarray data were analysed with the GeneXplain software version 3.0 [http://platform.genexplain.com/bioumlweb]. Here a total of 471 (293 up- and 178 down-) and 564 (353 up- and 211 down-) DEGs were determined after single and repeated treatment for 3 days, of which 132 genes were (89 up- and 43 down-) regulated in common amongst both datasets (see Figure 10A.1 and Table 2A). Besides, the 14 day repeated diclofenac treatment revealed 666 (455 up- and 211 down-) differentially expressed genes (DEGs) of which 74 were common when data from day 1, 3 and 14 were compared (Figure 10A.2 and Table 2B). Next, a heat map was generated using the average-linkage hierarchical clustering and the Euclidean distance algorithm. As shown in Figure 10B several genes were regulated in common coding for inflammatory, immune, stress and acute-phase response. Both of the softwares employed, i.e. GeneXplain and GenPlex informed on the significant regulation of the inflammatory, immune and stress response genes (Table S2), even though the total number of DEGs differed when the Welch and/or hypergeometric statistical test were applied.

**Table 2A:** Commonly regulated immune, inflammatory and stress response genes after single and repeated diclofenac treatment for 3 days

ID	Gene description	Fold Change	
		Day 1	Day 3
**Inflammatory and immune response**			
Apcs*	Serum amyloid p-component	4.28	3.92
Ccl6	Chemokine (c-c motif) ligand 6	2.99	1.68
Chi3l3*	Chitinase 3-like 3	3.9	3.89
Ctsc	Cathepsin c	1.95	2.40
Cxcl1*	Chemokine (c-x-c motif) ligand 1	17.36	19.10
Cxcl13*	Chemokine (c-x-c motif) ligand 13	1.98	2.02
Hp*	Haptoglobin	1.43	1.57
Ifitm2	Interferon induced transmembrane protein 2	1.67	1.51
Il33	Interleukin 33	1.74	1.54
Itih4*	Inter alpha-trypsin inhibitor, heavy chain 4	1.51	1.55
Lbp*	Lipopolysaccharide binding protein	1.88	1.67
Lcn2	Lipocalin 2	22.49	18.37
Marco	Macrophage receptor with collagenous structure	4.00	1.98
Nfkbiz*	Nuclear factor of kappa light polypeptide gene enhancer in b-cells inhibitor, zeta	2.90	1.69
Orm1*	Orosomucoid 1	1.72	1.52
Orm2*	Orosomucoid 2	8.46	10.83
Orm3	Orosomucoid 3	9.01	3.48
Prg4	Proteoglycan 4 (megakaryocyte stimulating factor, articular superficial zone protein)	4.18	2.74
S100a8*	S100 calcium binding protein a8 (calgranulin a)	14.02	7.46
S100a9*	S100 calcium binding protein a9 (calgranulin b)	8.54	4.13
Saa1*	Serum amyloid a 1	4.58	9.97
Saa2*	Serum amyloid a 2	9.83	35.20
Saa3*	Serum amyloid a 3	6.89	4.39
Saa4*	Serum amyloid a 4	1.78	2.18
Serpina3n*	Serine (or cysteine) peptidase inhibitor, clade a, member 3n	4.60	3.24
Stat3*	Signal transducer and activator of transcription 3	1.93	2.08
Tfrc*	Transferrin receptor	1.77	1.85
Ubc*	Ubiquitin c	1.74	−1.47
Vcam1	Vascular cell adhesion molecule 1	1.46	1.84
**Stress response**			
Clec2h	C-type lectin domain family 2, member h	−2.60	−2.47
Fabp5	Fatty acid binding protein 5, epidermal	3.08	1.68
Hspa1a	Heat shock protein 1a	1.71	−1.45
Icam1	Intercellular adhesion molecule 1	1.75	1.85
Lepr	Leptin receptor	2.00	−1.66
Plac8	Placenta-specific 8	2.00	1.68
Ptpn1	Protein tyrosine phosphatase, non-receptor type 1	1.59	1.62
Scara5	Scavenger receptor class a, member 5 (putative)	4.11	2.54
Socs3	Suppressor of cytokine signaling 3	3.05	3.43
**Regulation of Cell death**			
Gas1	Growth arrest specific 1	−1.58	−1.81
Litaf	Lps-induced tn factor	1.87	2.06
Phlda1	Pleckstrin homology-like domain, family a, member 1	−1.72	−1.57
Tsc22d1	Tsc22 domain family, member 1	−1.67	−1.55
**Drug metabolism and detoxification pathways**			
Aldh18a1	Aldehyde dehydrogenase 18 family, member a1	2.37	1.64
Ces1d	Carboxylesterase 1d	−1.87	−2.88
Ces1e	Carboxylesterase 1e	−1.76	−1.55
Cyp26a1	Cytochrome p450, family 26, subfamily a, polypeptide 1	−2.23	−2.71
Cyp3a13	Cytochrome p450, family 3, subfamily a, polypeptide 13	1.75	1.57
Cyb561	Cytochrome b-561	4.26	2.79
Gstt3	Glutathione s-transferase, theta 3	−1.78	1.49
Hsd3b5	Hydroxy-delta-5-steroid dehydrogenase, 3 beta- and steroid delta-isomerase 5	−2.42	−1.63
Qsox1	Quiescin q6 sulfhydryl oxidase 1	1.88	1.58
Sqle	Squalene epoxidase	1.90	2.42
Steap4	Steap family member 4	3.02	4.81
Ugt2b38	UDP glucuronosyltransferase 2 family, polypeptide B38	−1.53	−2.85
Upp2	Uridine phosphorylase 2	−1.61	−1.95
**Cellular homeostasis**			
Ibtk	Inhibitor of bruton agammaglobulinemia tyrosine kinase	1.82	1.61
Mt1	Metallothionein 1	2.45	2.06
Mt2	Metallothionein 2	2.61	2.19
Pdilt	Protein disulfide isomerase-like, testis expressed	−1.71	−1.59
**GPCR protein signaling pathway**			
Gnat1	Guanine nucleotide binding protein, alpha transducing 1	2.90	3.20
Lpar4	Lysophosphatidic acid receptor 4	−1.53	1.44
Ptgfr	Prostaglandin f receptor	−1.53	1.63
Sucnr1	Succinate receptor 1	−2.24	−1.67
**Jak-Stat signalling pathway**			
Csf2rb	Colony stimulating factor 2 receptor, beta, low-affinity (granulocyte-macrophage)	2.71	1.59
Csf2rb2	Colony stimulating factor 2 receptor, beta 2, low-affinity (granulocyte-macrophage)	2.44	1.52
Il13ra1	Interleukin 13 receptor, alpha 1	2.30	1.88
Lifr	Leukemia inhibitory factor receptor	−1.60	−1.76
Spry4	Sprouty homolog 4 (drosophila)	−1.55	−2.74
**Transporter activity**			
Atp11a	Atpase, class vi, type 11a	2.83	1.82
Fabp2	Fatty acid binding protein 2, intestinal	−1.77	−1.81
Mup4	Major urinary protein 4	−1.60	−1.53
Nipal1	Nipa-like domain containing 1	4.26	2.05
Slc16a5	Solute carrier family 16 (monocarboxylic acid transporters), member 5	1.56	1.53
Slc25a30	Solute carrier family 25, member 30	−1.75	−2.10
Slc3a1	Solute carrier family 3, member 1	2.60	2.24
Slc41a2	Solute carrier family 41, member 2	3.58	2.74
Slco1a1	Solute carrier organic anion transporter family, member 1a1	−1.77	−1.76
Slco1a4	Solute carrier organic anion transporter family, member 1a4	−1.50	−1.43
Timm8a2	Translocase of inner mitochondrial membrane 8 homolog a2 (yeast)	1.54	−1.43
**Metabolic processes**			
4930444A02Rik	Riken cdna 4930444a02 gene	1.59	1.43
Acpp	Acid phosphatase, prostate	2.45	1.77
Adck3	Aarf domain containing kinase 3	−1.92	−1.53
Agxt2l1	Alanine-glyoxylate aminotransferase 2-like 1	−1.79	−2.18
Arid5b	At rich interactive domain 5b (mrf1-like)	−2.04	−1.63
B3galt1	Udp-gal:betaglcnac beta 1,3-galactosyltransferase, polypeptide 1	3.53	7.23
BC048546	Cdna sequence bc048546	2.42	1.67
Car1	Carbonic anhydrase 1	−1.80	−1.46
Car13	Carbonic anhydrase 13	−1.67	1.49
Ctsj	Cathepsin j	3.68	1.50
Eif1a	Eukaryotic translation initiation factor 1a	1.97	1.69
Fgl1	Fibrinogen-like protein 1	2.17	1.96
Hes6	Hairy and enhancer of split 6 (drosophila)	−1.61	−1.61
Hs6st2	Heparan sulfate 6-o-sulfotransferase 2	1.42	1.47
Inmt	Indolethylamine n-methyltransferase	−1.79	−1.52
Isyna1	Myo-inositol 1-phosphate synthase a1	5.21	1.86
Itih3	Inter-alpha trypsin inhibitor, heavy chain 3	1.81	1.64
Lass6	Lag1 homolog, ceramide synthase 6	2.24	2.68
Ly6e	Lymphocyte antigen 6 complex, locus e	1.58	1.69
Mme	Membrane metallo endopeptidase	−2.14	−1.54
Mmp16	Matrix metallopeptidase 16	1.58	1.84
Nnmt	Nicotinamide n-methyltransferase	2.47	1.81
Nova1	Neuro-oncological ventral antigen 1	−1.55	1.42
Prtn3	Proteinase 3	8.84	3.72
Rpl39l	Ribosomal protein l39-like	1.69	1.45
Stk31	Serine threonine kinase 31	−1.47	1.51
Tgm1	Transglutaminase 1, k polypeptide	1.63	1.93
Tspan8	Tetraspanin 8	1.75	1.63
**Regulation of signalling**			
D0H4S114	Dna segment, human d4s114	−2.30	−1.96
Lrg1	Leucine-rich alpha-2-glycoprotein 1	2.30	2.26
Tcl1b3	T-cell leukemia/lymphoma 1b, 3	2.61	1.80
Tifa	Traf-interacting protein with forkhead-associated domain	1.86	3.52
Tspan4	Tetraspanin 4	1.68	1.92
Vwce	Von Willebrand factor C and EGF domains	−2.01	−1.72
**Binding activity**			
Cpne8	Copine viii	3.44	2.48
Dnajc12	Dnaj (hsp40) homolog, subfamily c, member 12	3.29	2.70
Edil3	Egf-like repeats and discoidin i-like domains 3	−1.56	−1.46
Ifitm6	Interferon induced transmembrane protein 6	3.36	1.69
Klrb1b	Killer cell lectin-like receptor subfamily b member 1b	1.72	1.43
Mpeg1	Macrophage expressed gene 1	2.37	1.56
Opcml	Opioid binding protein/cell adhesion molecule-like	1.45	1.47
Rab11fip3	Rab11 family interacting protein 3 (class ii)	1.52	1.42
Rpap3	Rna polymerase ii associated protein 3	2.59	1.69
Serpina10	Serine (or cysteine) peptidase inhibitor, clade a (alpha-1 antiproteinase, antitrypsin), member 10	1.95	1.91
Snx10	Sorting nexin 10	1.78	1.95
Tmem176a	Transmembrane protein 176a	1.79	1.52
**Cellular development and differentiation**			
Fam55d	Family with sequence similarity 55, member d	1.56	1.51
Fndc3b	Fibronectin type iii domain containing 3b	2.99	2.04
Lce1d	Late cornified envelope 1d	−1.84	1.68
Mtap7d1	Microtubule-associated protein 7 domain containing 1	1.61	1.77
Phyhipl	Phytanoyl-coa hydroxylase interacting protein-like	−1.49	1.92
Sema4b	Sema domain, immunoglobulin domain (ig), transmembrane domain (tm) and short cytoplasmic domain, (semaphorin) 4b	1.44	1.67
Shisa6	Shisa homolog 6 (xenopus laevis)	−1.45	−1.62

Mice were treated with 30 mg/kg diclofenac once daily for up to 3 days. Whole genome microarray studies were performed and analyzed as detailed in the Material and Method section. 132 statistically significantly regulated genes (*p* < 0.01) were in common when single and repeated treatment groups were compared.

* The marked genes are also involved in stress response.

**Table 2B:** Commonly regulated immune, inflammatory and stress response genes after single and repeated diclofenac treatment for 14 days

ID	Gene description	Fold Change		
		Day 1	Day 3	Day 14
**Inflammation and immune response**				
Apcs*	Serum amyloid p-component	4.28	3.92	6.77
Ccl6	Chemokine (C-C motif) ligand 6	2.99	1.68	3.66
Cxcl1*	Chemokine (C-X-C motif) ligand 1	17.36	19.10	25.73
Cxcl13*	Chemokine (C-X-C motif) ligand 13	1.98	2.02	14.62
Hp*	Haptoglobin	1.43	1.57	1.44
Il33	Interleukin 33	1.74	1.54	4.41
Itih4*	Inter alpha-trypsin inhibitor, heavy chain 4	1.51	1.55	2.18
Lcn2*	Lipocalin 2	22.49	18.37	12.11
Lbp*	Lipopolysaccharide binding protein	1.88	1.67	−1.69
Marco*	Macrophage receptor with collagenous structure	4.00	1.98	7.20
Nfkbiz*	Nuclear factor of kappa light polypeptide gene enhancer in B-cells inhibitor, zeta	2.90	1.69	3.33
Orm1*	Orosomucoid 1	1.72	1.52	2.28
Orm2*	Orosomucoid 2	8.46	10.83	8.19
Prg4	Proteoglycan 4 (megakaryocyte stimulating factor, articular superficial zone protein)	4.18	2.74	4.91
S100a9*	S100 calcium binding protein A9 (calgranulin B)	8.54	4.13	10.66
Saa3*	Serum amyloid A 3	6.89	4.39	14.33
Saa4*	Serum amyloid A 4	1.78	2.18	2.30
Serpina3n*	Serine (or cysteine) peptidase inhibitor, clade A, member 3N	4.60	3.24	4.65
Stat3*	Signal transducer and activator of transcription 3	1.93	2.08	1.99
Tfrc*	Transferrin receptor	1.77	1.85	2.72
**Stress response**				
Hspa1a	Heat shock protein 1A	1.71	−1.45	1.68
Icam1	Intercellular adhesion molecule 1	1.75	1.85	2.01
Plac8	Placenta-specific 8	2.00	1.68	2.29
Ptpn1	Protein tyrosine phosphatase, non-receptor type 1	1.59	1.62	1.65
Socs3	Suppressor of cytokine signaling 3	3.05	3.43	7.78
**Drug metabolism and detoxification pathways**				
Ces1d	Carboxylesterase 1D	−1.87	−2.88	−1.97
Ces1e	Carboxylesterase 1E	−1.76	−1.55	−1.87
Cyb561	Cytochrome b-561	4.26	2.79	4.42
Hsd3b5	Hydroxy-delta-5-steroid dehydrogenase, 3 beta- and steroid delta-isomerase 5	−2.42	−1.63	−1.43
Mt1	Metallothionein 1	2.45	2.06	1.83
Mt2	Metallothionein 2	2.61	2.19	2.21
Qsox1	Quiescin Q6 sulfhydryl oxidase 1	1.88	1.58	2.15
Sqle	Squalene epoxidase	1.90	2.42	6.79
Steap4	STEAP family member 4	3.02	4.81	14.58
Upp2	Uridine phosphorylase 2	−1.61	−1.95	−1.56
**Transporter activity**				
Atp11a	ATPase, class VI, type 11A	2.83	1.82	5.46
Fabp2	Fatty acid binding protein 2, intestinal	−1.77	−1.81	−1.66
Slc3a1	Solute carrier family 3, member 1	2.60	2.24	3.76
Slc41a2	Solute carrier family 41, member 2	3.58	2.74	7.28
Slco1a1	Solute carrier organic anion transporter family, member 1a1	−1.77	−1.76	−1.70
Slco1a4	Solute carrier organic anion transporter family, member 1a4	−1.50	−1.43	−1.54
Slc25a30	Solute carrier family 25, member 30	−1.75	−2.10	−1.64
**Regulation of Cell death**				
Gas1	Growth arrest specific 1	−1.58	−1.81	−1.51
Tsc22d1	TSC22 domain family, member 1	−1.67	−1.55	−1.43
**Response to cytokine stimulus**				
Il13ra1	Interleukin 13 receptor, alpha 1	2.30	1.88	2.56
Mme	Membrane metallo endopeptidase	−2.14	−1.54	−1.81
**Metabolic processes**				
Acpp	Acid phosphatase, prostate	2.45	1.77	3.03
Agxt2l1	Alanine-glyoxylate aminotransferase 2-like 1	−1.79	−2.18	−3.16
B3galt1	UDP-Gal:betaGlcNAc beta 1,3-galactosyltransferase, polypeptide 1	3.53	7.23	8.21
Car1	Carbonic anhydrase 1	−1.80	−1.46	−1.53
Ctsj	Cathepsin J	3.68	1.50	1.89
Eif1a	Eukaryotic translation initiation factor 1A	1.97	1.69	1.55
Fgl1	Fibrinogen-like protein 1	2.17	1.96	2.89
Inmt	Indolethylamine N-methyltransferase	−1.79	−1.52	−2.38
Isyna1	Myo-inositol 1-phosphate synthase A1	5.21	1.86	2.76
Itih3	Inter-alpha trypsin inhibitor, heavy chain 3	1.81	1.64	1.87
Ly6e	Lymphocyte antigen 6 complex, locus E	1.58	1.69	2.40
Prtn3	Proteinase 3	8.84	3.72	2.67
Stk31	Serine threonine kinase 31	−1.47	1.51	1.57
Sucnr1	Succinate receptor 1	−2.24	−1.67	−1.58
Tgm1	Transglutaminase 1, K polypeptide	1.63	1.93	2.21
**Regulation of signalling**				
Lrg1	Leucine-rich alpha-2-glycoprotein 1	2.30	2.26	2.27
Tcl1b3	T-cell leukemia/lymphoma 1B, 3	2.61	1.80	2.10
Tifa	TRAF-interacting protein with forkhead-associated domain	1.86	3.52	15.81
Tspan4	Tetraspanin 4	1.68	1.92	1.68
**Binding activity**				
Cpne8	Copine VIII	3.44	2.48	6.19
Dnajc12	DnaJ (Hsp40) homolog, subfamily C, member 12	3.29	2.70	2.09
Ifitm6	Interferon induced transmembrane protein 6	3.36	1.69	2.94
Mpeg1	Macrophage expressed gene 1	2.37	1.56	1.70
Rpap3	RNA polymerase II associated protein 3	2.59	1.69	2.45
Serpina10	Serine (or cysteine) peptidase inhibitor, clade A (alpha-1 antiproteinase, antitrypsin), member 10	1.95	1.91	3.05
Snx10	Sorting nexin 10	1.78	1.95	2.50
Tmem176a	Transmembrane protein 176A	1.79	1.52	2.21
**Cellular development and differentiation**				
Fndc3b	Fibronectin type III domain containing 3B	2.99	2.04	2.28

Mice were treated with 30 mg/kg diclofenac once daily for up to 14 days. Whole genome microarray studies were performed and analyzed as detailed in the Material and Method section. 74 statistically significantly regulated genes (*p* < 0.01) were in common when single and repeated treatment groups were compared.

* The marked genes are also involved in stress response.

### Regulation of transporters and drug metabolism genes

As diclofenac is extensively metabolized we initially assessed the regulation of genes coding for CYP enzymes and transporters. The microarray study revealed *Cyp26a1*, *Cyp2a4*, *Cyp2a5*, *Cyp3a13*, *Cyp2b9*, *Cyp2c40* and *Cyp2c55* to be significantly regulated after single treatment. Likewise, repeated treatment for up to 14 days caused significant regulations of *Cyp7a1*, *Cyp39A1*, *Cyp2c39*, *Cyp3a44* and *Cyp3a13*. Note, *Cyp7a1* catalyses the hydroxylation of cholesterol during bile acid synthesis and was significantly up-regulated after repeated treatment; nonetheless genes coding for transporters were more frequently changed than those involved in drug metabolism and included major mitochondrial solute carriers (Table S3).

### Top bio-functions of genes influenced by diclofenac treatment

The GeneXplain and GenPlex software defined the top biological processes of DEGs with significant *p*-value (Tables S4–S6). Using the Ingenuity Pathway Analysis software and by applying the Fisher's exact test a functional enrichment analysis was performed and the statistically significant DEGs were categorized into diseases and disorders, molecular and cellular functions, physiological system, development and function (see Tables S7 and S8). Significantly enriched ontology terms were mainly associated with inflammatory response, gastrointestinal disease, organism injury/abnormalities and renal/urological disease after single treatment. Similarly, the genomic responses after repeated treatment involved inflammation, infectious and inflammatory disease as well as respiratory and hematological disorders with several of the inflammatory response genes being equally regulated after single and repeated treatment.

Furthermore, significantly enriched ontology terms related to molecular and cellular functions were cellular movement, molecular transport, cell death, cellular assembly/organization after single treatment, whereas cellular movement, cell-to-cell signalling/interaction, antigen presentation, lipid metabolism and molecular transport were top functions after repeated treatment.

In the category physiological system, development and function significantly enriched terms were liver development and function, immune cell trafficking and tissue development with DEGs being related to hepatic system development and hematopoiesis as well as tissue morphology after single and repeated treatment. Finally, common ontology terms involved the hematological system development and function, inflammatory response and immune cell trafficking.

### Canonical pathway analysis

Canonical pathway analysis was done with the molecular pathways tool available in the IPA library. After single treatment three metabolic pathways and seven signalling pathways were enriched and involved inositol phosphate, aminosugar, fructose and mannose metabolism. The signalling pathways were related to cellular immune responses, cytokine signalling, humoral immune responses, disease-specific pathway and the ingenuity toxicity list (Figure S3, Panel A). After repeated treatment the top 10 signalling pathways were concerned largely with apoptosis, cardiovascular signalling, cellular immune responses, cytokine signalling, disease-specific pathways, the ingenuity toxicity list, nuclear receptor signalling, and organismal growth development (Figure S3, Panel B).

Among the altered pathways, immune-related signalling was commonly affected and involved genes coding for *Orm1*, *Orm2*, *Apcs*, *Serpina3*, *Stat3*, and *Lbp*, along with *Saa2* and *Saa4*. Additionally, interleukin signalling appeared in the canonical pathway analysis and involved genes coding for *Stat3*, *Socs3*, *Il-33*, *Il1r1* and the chemokines *Cxcl3* and *Cxcl13*.

### Regulatory gene networks

Apart from the initial pathway analysis we searched for regulatory gene networks. This defined the growth hormone receptor (*Ghr*), leptin receptor (*Lepr*) and protein tyrosine phosphatase, non-receptor type 2 (*Ptpn2*) as master regulators; their associated networks accounted for 140, 142 and 146 of significantly regulated DEGs or > 57% of total DEGs, respectively, after single treatment. Similarly, after repeated treatment > 50% of DEGs were connected in networks that consisted of the master regulators leptin receptor (*Lepr*), selectin (*Sele*) and suppressor of cytokine signalling 3 (*Socs3*). Moreover, common DEGs after single and repeated treatment for 3 days were grouped into networks with the leptin receptor, lipocaline 2 (*Lcn2*) and *Nfkb* as central acting molecules and accounted for 50% of significantly regulated DEGs. After 14 days of treatment the serum amyloid P-component (*Apcs*), CD44 antigen (*Cd44*) and cytotoxic T lymphocyte-associated protein 4 (*Ctla4*) were defined as master regulators and involved 31% of DEGs in their associated networks. Note, the genes coding for the master regulators were likewise significantly regulated (see Table 3).

**Table 3:** Master regulator genes after single and repeated diclofenac treatment for 14 days

Master regulatory genes	No of genes		Score	FDR	*Z*-score	Average Fold change	Rank sum
	Total No of genes in the network	Statistically significant DEGs					
**After single dose (day 1)**							
Leptin receptor (Lepr)	248	142	0.47394	0.011	2.54686	1.91	15
Growth hormone receptor (Ghr)	247	140	0.42311	0.018	2.88692	−1.48	18
Protein tyrosine phosphatase, non-receptor type 2 (Ptpn2)	253	146	0.42122	0.029	2.22294	1.57	27
**After repeated treatment (day 3)**							
Leptin receptor (Lepr)	254	145	0.44159	0.047	1.88705	−1.66	14
Selectin, endothelial cell (Sele)	262	153	0.44818	0.05	1.95103	1.61	12
Suppressor of cytokine signallingsignaling 3 (Socs3)	287	158	0.59558	0.019	1.78924	3.43	11
**Common DEGs after single (day 1) and repeated treatment (day 3)**							
Leptin receptor (Lepr)	68	33	0.62653	0.001	1.93479		17
Lipocalin 2 (Lcn2)	78	34	0.44549	0.015	1.96325	22.62	42
Nuclear factor of kappa light polypeptide gene enhancer in B cells inhibitor, zeta (Nfkbiz)	70	34	0.4655	0.006	2.13014	2.91	31
**After repeated treatment (day 14)**							
Serum amyloid P-component (Apcs)	144	88	0.43611	0.047	1.76832	6.77	2
Cytotoxic T-lymphocyte-associated protein 4 (Ctla4)	161	94	0.55188	0.048	1.28056	2.17	3
CD44 antigen (Cd44)	172	96	0.6616	0.046	1.18176	1.77	3

Given is a summary of master regulatory networks with the number of total interacting genes and DEGs within the constructed networks, network score, *Z*-score and average fold change.

Note: *Z*- Score > 1 and Score > 0.2 was set to select statistically significant master regulators.

To search for cross-talk amongst individual regulatory gene networks an integrated network was constructed and 31%, 28% and 15% of DEGs were commonly regulated after single and repeated treatment, respectively (see Figures S4–S7). Intriguingly, the master regulatory molecules *Lepr*, *Lcn2*, *Nfkbiz*, *Socs3*, *Apcs* and *Ctla4* were also significantly regulated in the fused network amongst all treatments.

### Validation studies by qRT-PCR, western blotting and immunohistochemistry

**Figure 11:**
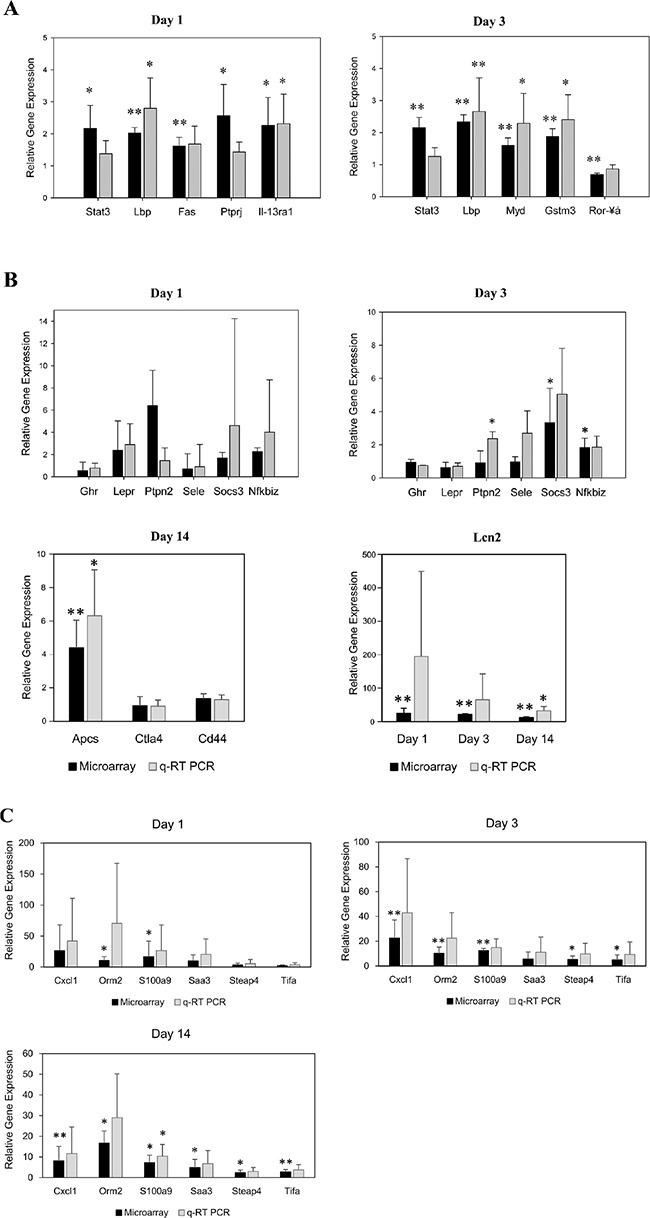
Microarray data validation by real time quantitative PCR (**A**) Validation of microarray data by quantitative real-time PCR of randomly selected genes. The *y*-axis indicates the relative fold change in expression (diclofenac-treated *vs*. saline-treated controls). Data are means ± SD (*n* = 3). *P < 0.05, **P < 0.01. (**B**) Validation of master regulators by quantitative real-time PCR. The *y*-axis indicates the relative fold change in expression (diclofenac-treated *vs*. controls). Data are means ± SD (*n* = 3). *P < 0.05, **P < 0.01. (**C**) Validation of cytokines by quantitative real-time PCR. The *y*-axis indicates the relative fold change in expression (diclofenac-treated *vs*. controls). Data are means ± SD (*n* = 3). *P < 0.05, **P < 0.01.

Initially, a total of eight genes were selected with minor to moderate changes in gene expression to confirm their regulation by an independent method and except for *Stat3*, *Fas* and *Ror-a* there was agreement amongst both platforms in determining significant differences between control and diclofenac treated animals (Figure 11A). The regulation of genes coding for master regulators was also studied by qRT-PCR and the results are given in Figure 11B. Because of the variable responses amongst individual animals statistical significance could not be established even though some of the master regulators were > 10-fold induced and the coded protein were significantly up-regulated (see WB experiments below). Next regulation of cytokines was investigated by qRT-PCR (see Figure 11C and Table S9 for regulated cytokines and cluster of differentiation molecules found to be regulated by diclofenac) and trend wise the microarray and qRT-PCR data agreed; however, the data were more variable in qRT-PCR assays. Diclofenac treatment caused significant hepatic glycogen depletion (see hepatic PAS staining Figure 2B and 2C); we therefore studied regulation of genes involved in glycogenesis and carbohydrate metabolism. This revealed the genes hexokinase 1 (Hk1) and enolase 1 (Eno1) to be moderately but significantly regulated. Specifically, *Hk1* catalyses the production of glucose-6-phosphate, a rate limiting step in mitochondrial glycolysis as well as Eno1, which determines the conversion of 2-phosphoglycerate to phosphoenolpyruvate (see Figure S8).

**Figure 12:**
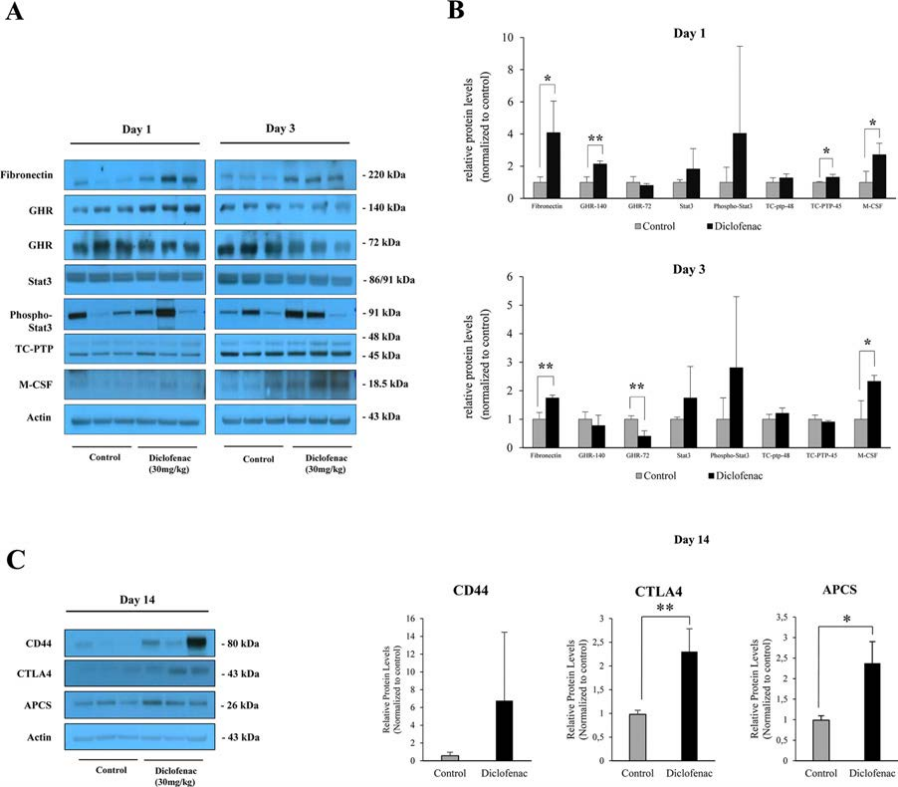
Western blotting of master regulatory proteins in liver extracts of diclofenac treated mice Depicted is the expression of master regulators after single and repeated treatment of mice for up to 14 days. (**A** and **C**) depict the Western immunoblotting results; the histograms in (**B**) represents densitometric scans of the immunoblots relative to the vehicle control. Data are expressed as mean and standard deviation. *P < 0.05, **P < 0.01.

Next Western immunoblotting studies were performed to reveal significant regulation of fibronectin, growth hormone receptor (oppositely on day 1 and 3) and M-CSF (Figure 12A and 12B) and although pSTAT3 expression was highly induced among individual animals it failed statistical significance testing. The extremely large scattering of phospho-Stat3 might be due to some oscillation in the phosphorylation of Stat3 with animals not being synchronised in the circadian rhythm. After single diclofenac treatment, a small but statistically significant induction of TC-PTP was observed (see Figure 12A and 12B) while Western blotting of master regulatory molecules after repeated diclofenac treatment for 14 days identified CD44, CTLA4 and APCS to be up-regulated; however only the latter two proteins reached statistical significance (Figure 12C).

The expression of master regulatory molecules was also studied by immunohistochemistry as detailed above.

### STRING PPI networks

**Figure 13:**
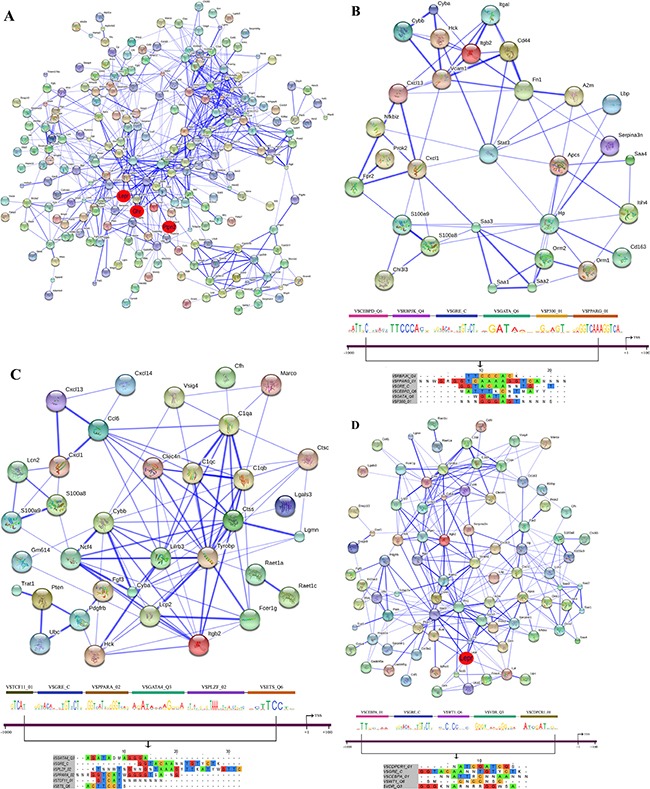
String protein interaction network and composite modules in livers of diclofenac treated mice after single treatment (**A**) Out of 471 DEGs a protein interaction network was constructed that consists of 225 DEGs and involved 749 PPIs. The red circle highlights the master regulatory genes; the strength of association amongst individual proteins is illustrated with the thickness of the blue line as defined in the STRING version 9.1. (**B**) Inflammatory response sub-network with its composite module of co-bound transcription factors at promoters of regulated genes. (**C**) Immune response sub-network with its composite module of co-bound transcription factors at promoters of regulated genes. (**D**) Stress response network with its composite module of co-bound transcription factors at promoters of regulated genes.

**Figure 14:**
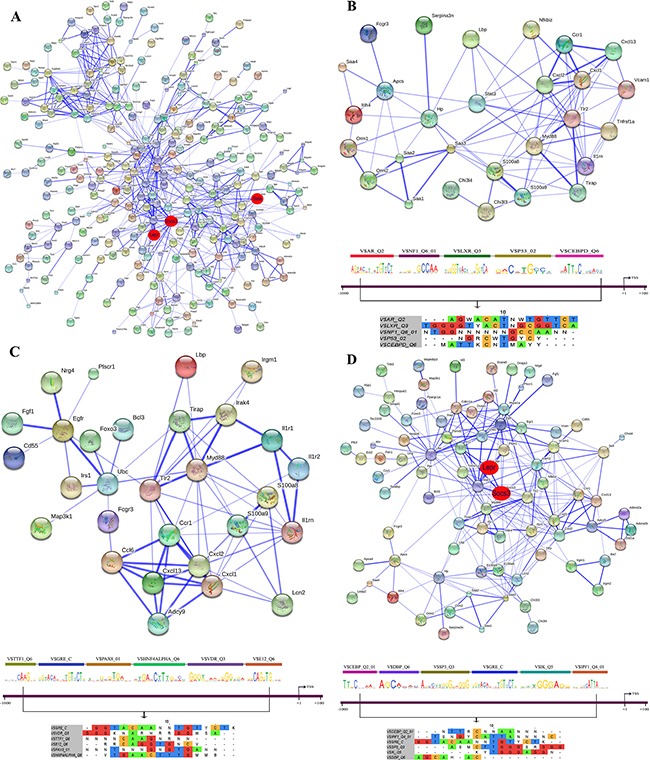
String protein interaction network and composite modules in livers of diclofenac treated mice after repeated treatment for 3 days (**A**) Out of 564 DEGs a protein interaction network was constructed that consisted of 277 DEGs and involved 687 PPIs. (**B**) Inflammatory response sub-network and its composite module. (**C**) Immune response sub-network and its composite module. (**D**) Stress response sub-network and its composite module. Please see Figure 13 for a description of the inferred association defined by the STRING version 9.1.

**Figure 15:**
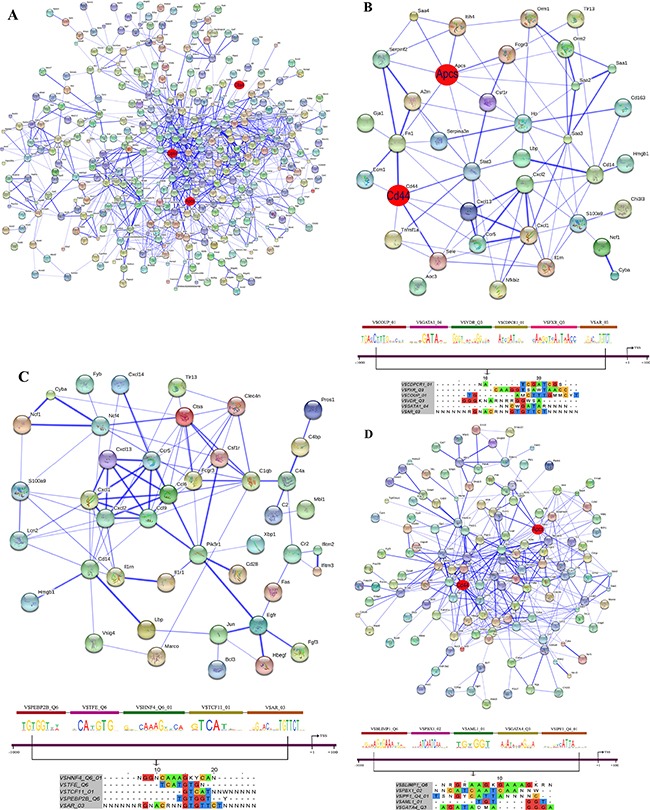
String protein interaction network and composite modules in livers of diclofenac treated mice after repeated treatment for 14 days (**A**) Out of 666 DEGs a protein interaction network was constructed that consists of 348 DEGs and involved 1043 PPI. (**B**) Inflammatory response sub-network and its composite module. (**C**) Immune response sub-network and its composite module. (**D**) Stress response sub-network and its composite module. Please see Figure 13 for a description of the inferred association defined by the STRING version 9.1.

Protein-protein interactions (PPI) were determined with the String software version 9.1. Overall, 48% of DEGs had proven PPIs after single and/or repeated treatment and therefore interact with each other. As diclofenac treatment influenced the expression of genes associated with inflammation, immune and stress response (see Table S2) individual PPI networks were constructed; as shown in Figures 13A–15A the total numbers of PPIs were 749, 687, 1043 after single and repeated treatment for 3 and 14 days, respectively. Furthermore, the numbers of PPIs within the inflammation, immune and stress response networks were 75, 107 and 282 after single (see Figure 13B–13D) and 89, 74, 256 after repeated treatment for 3 days (see Figure 14B–14D). Likewise, diclofenac treatment for 14 days was associated with 92, 96 and 413 PPIs, respectively (see Figure 15B–15D).

### Co-occupancy of transcription factor binding sites in promoters of regulated genes

To define molecular circuits in inflammation the involvement of different transcription factor at gene specific promoters of inflammatory, immune and stress response genes was investigated and included computational analysis using position weight matrices available in the TRANSFAC database in addition to entrained genetic algorithms (see Table S10–S12). After single treatment and among 35 inflammatory responsive genes 28, 27, 34, 29, 23 and 17 were significantly enriched for *Cebpd, Rbpjk, Gre, Gata, P300* and *Pparg* transcription factor binding sites, respectively (see Figure 13B) whereas at gene specific promoters of *Orm2*, *Cxcl1*, *S100a8* and *Hck*, the entire composite module could be fitted. Similarly, after repeated diclofenac treatment for 3 days the composite module encompassed 37 inflammatory response genes with 25, 14, 17, 8, 32 and 14 genes being significantly enriched for *Ar*, *Nf1*, *Lxr*, *P53* and *Cebpd* transcription factor binding sites, respectively (Figure 14B). Next the composite module for common DEGs was computed and revealed significantly enriched *Gata*, *Gfi1*, *Nf1*, *Cebpa* and *Dr1* transcription factor binding sites (Figure S9). Previous research already identified C/EBP proteins to play a major role in the inflammatory response. After repeated treatment for 14 days inflammatory response genes involved the *Coup1*, *Gata1*, *Vdr*, *Cdpcr1*, *Fxr* and *Ar* transcription factors (Figure 15B). Conversely, promoters of the immune response genes were significantly enriched for *Tcf11*, *Gre*, *Ppara*, *Gata4*, *Plzf* and *Ets* TF binding sites after single (Figure 13C) and *Ttf1*, *Gre*, *Pax8*, *Hnfalpha*, *Vdr* and *E12* after repeated treatment for 3 days (Figure 14C). Likewise, after 14 days of treatment the composite module consisted of the transcription factors *Pebp2b* (*Cbfb*), *Tfe*, *Hnf4*, *Tcf11* and *Ar* (Figure 15C).

When promoters of stress response regulated genes were interrogated the composite modules consisted of *Cebpa*, *Gre*, *Wt1*, *Vdr* and *Cdpcr1* after single (see Figure 13D) and *Cebp*, *Dbp*, *Sp3*, *Gre*, *Ik* and *Ipf1* after repeated treatment for 3 days (Figure 14D). After 14 days of treatment a composite module was constructed and consisted of *Blimp1*, *Pbx1*, *Aml1*, *Gata4* and *Ipf1* transcription factors (Figure 15D). Note, the frequency of transcription factor binding sites in promoters of inflammatory, immune and stress response genes is given in Tables S13–S15.

### Studies with mouse hepatocyte cultures

Whole genome gene expression profiling of diclofenac treated primary mouse hepatocytes cultures revealed 2663 (1371 up- and 1292 down-) DEGs with the fold change of > 2 and *p*-value cut off of < 0.01. Furthermore, the comparison of *in vitro* and *in vivo* data (day 1, 3 and 14) defined 20 genes to be regulated in common (see Figure S10), however *Cxcl1, Dnajc12, Hp, Hsd3b5, Icam1, Il-33, Ly6e, Marco, Nfkbiz, Saa4, Serpina10, Steap4* and *Tmem176a* were oppositely regulated when compared to *in vivo* findings (Table S16).

## DISCUSSION

Diclofenac is a commonly prescribed NSAID for the treatment of pain and inflammation; however, its use is associated with adverse drug reaction most notable increased risk for arterial thrombotic event that prompted new safety advice by the European Medicines Agency in 2013. Apart from cardiovascular complications its use is also associated with significant gastrointestinal toxicity and drug-induced liver injury (DILI) of different grades including rare but life-threatening acute liver failures that required liver transplantation as determined in a recently published retrospective study involving 52 centres across 7 European countries [14]. A newly published study on the incidence, presentation and outcome in patients of the general population of Iceland also revealed diclofenac to rank second amongst drugs causing DILI [15] while histopathology findings of clinical cases are suggestive for an immune allergic reaction type in diclofenac DILI cases.

In an effort to define immune-mediated mechanisms of hepatotoxicity we investigated genomic responses in livers of mice after single and repeated diclofenac treatment. This revealed key molecular processes whereby diclofenac elicits an inflammatory, acute phase and immune response. Our findings agree with the general notion on diclofenac hepatotoxicity whereby reactive metabolites and immune mediated mechanisms lead to liver injury [16].

Specifically, the potential to cause liver injury in mice was investigated at diclofenac doses of 50, 80, and 120 mg/kg with serum transaminase activity being significantly increased at 80 and 120 mg/kg after 24 hours of treatment [10]. In this study diclofenac-induced liver injury was associated with regulation of *Il-1Δ* and *in vivo* blocking/neutralization of *Il-1Δ* activity with an inhibitory antibody ameliorated liver injury to suggest *IL-1β* to be involved in the pathogenesis of liver injury. In the present study and at the lower dose of 30 mg/kg body weight *Il-1Δ* was also significantly up-regulated (nearly 3-fold) after 14 days of treatment (see Table S9). At this dose significant changes in serum ALT, AST and ALP activities were observed, however 150 mg/kg induced mortality and acute liver failure. Therefore, our study evidences acute lethality at significantly lower doses to the previously reported LD50 of 345 mg/kg [TOXNET Database: http://chem.sis.nlm.nih.gov/chemidplus/rn/15307-86-5].

Owing to its extensive metabolism we initially assessed regulation of genes coding for drug metabolism and transport, however observed only minor, yet statistically significant changes after diclofenac treatment (see Table S3). Furthermore, the expression of *Sod2* was decreased in diclofenac-treated livers and this enzyme transforms toxic superoxide into hydrogen peroxide and oxygen in mitochondria. Decreased expression levels of *Sod2* are associated with oxidative stress as was shown in a previous performed hepatocyte cell culture study where production of reactive oxygen species (ROS) increased upon diclofenac treatment [5].

Diclofenac treatment induced innate and adaptive immune responses [10, 17–19] as demonstrated in the present study by the regulation of various chemokines *Cxcl1*, *Cxcl2*, *Cxcl13*, *Xcl1*, *Ccl6*, *Tnfaip8l2*, *Tnfsf12/13*, *Il-7*, *Il-15* and cytokine receptors *Il1r1*, *Il4ra*, *Fas* (see Table S2). Specifically, *Cxcl1*, *Cxcl2*, and *Cxcl13* facilitate recruitment of neutrophils, leukocytes, and B lymphocytes to sites of injury whereas *Xcl1* and *Ccl6* support differentiation of myeloid cells and CD8^+^ T cells [20, 21]. Increased expression of *Il-7* by hepatocytes controls T cell responses [21] while *Il-15* regulates the activation and proliferation of T cells and natural killer (NK) cells [22]. Additionally, *Il-33* was up-regulated and this member of the *Il-1* superfamily stimulates production of type 2 cytokines by T helper cells [23]. Thus, diclofenac treatment resulted in an increased expression of cytokines to influence T cell differentiation. According to the danger hypothesis, a danger signal is induced by immune responses to antigens as well as the interaction of pro-inflammatory cytokines and polarizing cytokines [24] and in the case of diclofenac involved regulation of *Tlr2*, *Tlr13* and the apoptosis related genes *Fas*, *Tnfrsf1a*, *Myd88*, and *Nfkbiz*. The *Chi3l3* gene was highly expressed, and this protein is known to be released from macrophages during inflammation. Diclofenac also induced *S100a8* and *S100a9* transcript expression and increased levels of the proteins hallmark numerous pathological conditions associated with inflammation [25].

Furthermore, the expression of acute phase response genes was up-regulated in response to diclofenac treatment. Acute phase proteins are produced by hepatocytes and controlled by cytokines such as *Il-1β*, *Tnf-α*, *Ifn*γ, and *Il*-6 [26]. According to the study of Yano *et al.* 2012 [10] expression of *Il-1* is highest at 3 h, but expression of *Tnf*-α peaked at 24 h in diclofenac-treated mouse liver (80 mg/kg, *i.p*.) [11].

Additionally, *Stat3* is known to be a transcription factor activated by cytokines and growth factors and plays a key role in many cellular processes such as cell growth and apoptosis. In hepatocytes, *Stat3* is directly involved in the transcription of acute phase proteins by stimulating *Il-6* and interaction with *Nfkb1* [26]. In the present study, phosphorylated *Stat3* was highly induced in some of the treated animals (see Figure 12) and this protein controls transcription of *Socs3* which functions as an inhibitor of *Jak/Stat3* signalling [27]. Notably, *Socs3* supports differentiation of the Th17 and is implicated in autoimmune disease and a recent study reported that the expression of Th17 cell-mediated factors such as *ROR*-γt, and *Stat3* increased significantly in diclofenac-treated mouse liver [10].

Taken collectively, the release of proinflammatory cytokines by diclofenac stimulates cytokine receptor activation. Moreover, *Stat3*, which is activated by cytokine signalling, induces expression of acute phase proteins and differentiation of T cells and this feedback-loop is controlled by *Socs3* [28]. Expression of *Il4ra* and *IL13ra1* was also increased in response to diclofenac treatment; the coded proteins are known to activate *Jak/Stat6* signalling through binding of *Il-4* and *Il-13* [29]. Therefore, the Jak/Stat, PPAR, adipocytokine and chemokine signalling pathways were significantly influenced after single and repeated diclofenac treatment.

### Molecular circuits of inflammation

The network analysis defined several master regulators, i.e. the leptin receptor, *Ghr*, *Ptpn2*, *Socs3, Sele*, *Lcn2*, *Nfkbiz*, *Ctla4*, *Apcs* and *Cd44* after single and repeated diclofenac treatment. Four of the master regulators (*Lepr Ghr, Ptpn2 and Socs3*) directly interact with *Stat3* to significantly influence the constructed gene networks. As described above, *Stat3* is rapidly activated by various cytokines and growth factors including *Il-6*, EGF family members and hepatocyte growth factor as part of an immune response and inflammation [30–32]. This factor intercede regulation of several pro-inflammatory (*Mapks*, p38, *Jnk*, and *IκB* kinase) and anti-inflammatory (*Pi3k-Akt*) signalling cascades as had been observed in LPS treated mice [25, 33]. Another factor significantly up-regulated in response to diclofenac treatment was leptin and this 16-kDa adipokine plays a key role in energy intake to influence monocyte/macrophage-mediated responses during inflammation [34]. Several studies suggest leptin to be a pro-inflammatory molecule whereby the leptin receptor directly or indirectly modulates signalling pathways involved in kinase-induced phosphorylation by *Jak2/Stat3*, *Erbb2*, *Erk, Irsl* and *Rho/Rac* [35]. This cytokine interacts with *Socs3*, i.e. another master regulator of the repeated diclofenac treatment network; its interaction with *Stat3* constitutes a negative feedback loop on leptin receptor activity [36, 37]. Several of the pro-inflammatory cytokines found to be up-regulated in the present study also induce expression of *Socs3* and this protein is an important master regulator that can be activated by STAT and nuclear factor κΒ (NF κΒ)-mediated pathways [38].

The growth hormone receptor is another master regulator identified in the network analysis and plays a major role in response to tissue injury [39, 40]. Importantly, the growth hormone and leptin receptors are influenced by multiple intracellular signalling cascades, including Jak-Stat pathway [41] and chemokine signalling pathway found to be regulated in the present study (*Hck, Ncf1, Stat3, Nfkb1,* and *Cxcl1*) and are a part of the hepatic inflammatory response [42] to suppress hepatic *Ghr* signalling [43]. Specifically, GHR protein expression was reduced at day 3 of diclofenac treatment thus evidencing its degradation (see Figure 12).

Additionally, the protein tyrosine phosphatase (*Ptpn2*) was identified as a master regulator and it's regulation by diclofenac is of particular importance in the modulation of interferon gamma signal transduction as part of the inflammatory pathway [44]. In the present study Tc-PTP was modestly but significantly up-regulated as determined by qRT-PCR and immunoblotting. The present study also identified E-selectin as a master regulator whose decisive roles in placing leukocytes to the site of injury during inflammation has been documented [45]. The increased expression of *Lcn2* is of clinical significance and is associated with activation of neutrophils as seen in inflammation and oxidative stress conditions [46]. It is also strongly induced in hepatocytes and adipocytes, both *in vitro* and *in vivo* by pro-inflammatory cytokines, such as interleukin-1Δ and tumour necrosis factor alpha and was induced by diclofenac treatment [47].

Network analysis of 14 day repeated diclofenac treatment data revealed the cytotoxic T lymphocyte-associated antigen 4 (*Ctla4*) as master regulator; note, the coded protein is a key element in the immune system to induce immune tolerance and is one of the critical negative regulators of the T cell-mediated immune response. Furthermore, *Ctla4* gene polymorphism is a risk factor for drug induced liver injury [48] and is associated with autoimmune liver diseases including primary biliary cirrhosis [49]. *Ctla4*-mediated signal transduction was also reported to induce cell death in previously activated T cells and abnormal expression of *Ctla4* can be associated with varies pathologies including chronic immune diseases as well as malignancies [50]. Likewise, the serum amyloid P-component (*Sap* or *Apcs*) is a major acute phase protein that is synthesized in response to pro-inflammatory cytokines and binds to DNA to modulate immune responses [51]. It was reported that hepatic *Apcs* gene expression is enhanced during inflammation [52, 53].

Lastly, network analysis revealed *Cd44* as a master regulator which we found highly but variably regulated amongst individual animals (Figure 12). This membrane bound receptor functions in the regulation of several biological processes including adhesion, migration, invasion, survival and inflammation [54] and plays a role in a variety of inflammatory responses, including the induction of pro-inflammatory cytokines and the migration of macrophages and neutrophils [55, 56]. Binding of hyaluronic acid to *Cd44* promotes the interaction with a number of other cell surface proteins, for instance *Tlr4* and *Egfr*, and influences the activity of a variety of downstream protein kinase signalling pathways (MAP kinase and Akt pathways) [57]. It was also reported that *Cd44* provides a critical link between cellular metabolic changes and the development of inflammation as well as insulin resistance in liver [58].

### Co-occupancy of transcription factor binding sites

Regulatory gene networks were interrogated by analysing transcription factor binding sites at gene specific promoters. Eventually, composite modules were computed after single and repeated treatments. A notable finding is the vitamin D receptor (*Vdr*), i.e. a transcription factor that was significantly regulated in either composite module after single and repeated diclofenac treatment regimens. Several independent studies implicate *Vdr* in the inflammatory response [59–61] and in the innate and adaptive immune system [62–64] by regulating the production of inflammatory cytokines and inhibiting the proliferation of pro-inflammatory cells, both of which are crucial for the pathogenesis of inflammatory and autoimmune diseases. Moreover, *Vdr* polymorphisms are associated with risk and severity of liver diseases such as primary biliary cirrhosis, autoimmune hepatitis and hepatocellular carcinoma [65, 66].

Besides, the transcription factor and master regulator glucocorticoid receptor (*Nr3c1*) is strongly implicated in the regulation of inflammatory, immune and stress response genes after single and repeated diclofenac treatment with most of the inflammatory response genes being bona fide targets for *Gre* and *Nfkb* [67, 68].

Furthermore, the CCAAT/enhancer-binder protein (C/EBP) families of transcription factors (i.e. *Cebpd* and *Cebpa*) play an important role in the transcriptional regulation of inflammatory and stress response genes [69]; thus a complex interplay exists by which *Tnf-alpha*, *Il-1*, nuclear factor κB, activator protein-1, early growth response protein-1 and C/EBPs to intensify the inflammatory response [70] with *Cebp*Δ and d isoforms being up-regulated while *Cebpa* was down regulated in response to inflammation [33]. The co-occupancy TF analysis also revealed GATA factors to take part in the inflammatory and stress response after single and repeated diclofenac treatment. Among the GATA Zn-finger proteins isoforms 1 to 3 play major roles in the hematopoietic and immune system [71] whereas *Gata4* and *Gata6* participate in the control of liver-specific gene expression as was shown by Mwinyi et al. 2010 [72]. It was reported that *Gata2* and 3 engage in protein complexes with *Cebpa/CebpΔ* to jointly suppress adipocyte differentiation [73]. In addition, the liver-specific transcription factors *Hnf1a* and *Hnf4a* are part of the constructed inflammatory and immune composite modules and play a crucial role in the regulation of metabolism including bile acid, cholesterol and lipoprotein metabolism as well as glucose and fatty acid metabolism by maintaining hepatocyte differentiation [74–76]. During inflammation the metabolic competence of the liver is greatly affected and involves altered nuclear receptor activity, cross-talk amongst different cell types including macrophages, B and T cells, cytokine signalling and cytokine mediated activity of liver enriched transcription factors to influence liver regeneration, apoptosis, and liver-specific gene regulation as had been reviewed by us [74, 77, 78].

Moreover, repeated diclofenac treatment elicited regulation of the *P53* tumour suppressor. This transcription factor is activated in response to different cellular stresses including DNA damage and hypoxia and mediates a variety of anti-proliferative processes [79]. The interaction of p53 with the transcription co-activators p300 (see Figure 14B) and CREB-binding protein (CBP) enhances its ability to bind and activate transcription of target genes [80]. Likewise, *Ppar* is a part of the composite module constructed after single diclofenac treatment and the *Ppar* family transcription factors can be activated by numerous fatty-acid metabolites that are produced during the inflammatory response. *In vivo* and *in vitro* studies evidence PPAR signalling pathway to be significantly regulated in diclofenac treated mice [81] and *Ppary* is involved in negative regulation of *Il-6* mediated acute phase response in hepatocytes. The transcription factors *Ppary* and *Cebpa* are both inferred in the stress and inflammatory response composite modules after single diclofenac treatment and are known to promote adipocyte differentiation [82] with *Ppary* regulating the expression of a number of genes involved in peroxisomal and mitochondrial fatty acid β-oxidation. Similarly, *Lxr* has been characterized as regulator of macrophage inflammatory pathways and is a part of the constructed inflammatory response composite module after repeated diclofenac treatment for day 3.

Therefore, coordinate regulations of *Ppar* and *Lxr* transcription factors and glucocorticoid receptor have been proposed to integrate local and systemic responses to inflammation [83, 84]. Recent studies also highlight the role of chromatin higher order structure in the regulation of inflammatory gene expression and specifically the p300 co-activator protein. This histone acetylase functions through various interaction domains such as the RID, KIX, the interferon response binding domain and p53 with transcription factors defined in the inflammatory and immune response networks (see Figure 14B). A further member of the constructed composite module is *Rbp-j*, i.e. a DNA binding protein that plays a key role in signal transduction during myeloid cell differentiation by the Notch signalling pathway [85, 86].

The farnesoid X receptor (*Fxr*) was regulated by diclofenac and apart from its role in bile acid homeostasis it also functions in triglycerides, cholesterol and carbohydrate metabolism during the acute phase response [87]. It was shown earlier that pro-inflammatory cytokines induce *Fxr* activity during infection and inflammation, accompanied by abnormalities in lipid metabolism that are similar to those seen in common disorders, such as diabetes, obesity, and the metabolic syndrome [88, 89].

A further transcription factor of the inflammatory and immune response composite module with eminent importance in liver biology is the androgen receptor (*Ar*); altered *Ar* signalling is observed in various liver diseases including steatosis, cirrhosis and hepatocellular carcinoma [90] and was shown to play a suppressive role on liver gene expression during inflammation. In addition, exaggerated *Ar* activity may promote hepatocarcinogenesis via increased cellular oxidative stress, DNA damage/repair and cell apoptosis [91, 92].

Lastly, our network analysis of diclofenac induced immune response genes inferred regulation of the *Tcf11/Nrf1* transcription factor and the protein is known to influence expression of genes involved in glutathione (GSH) biosynthesis and other oxidative defence enzymes. Importantly, knockout of the gene in the liver of mice caused increased inflammation, apoptosis and spontaneous development of hepatic cancer [93]. The transcription factor *Tfe3* is part of day14 immune response composite module and plays a major role in activation of the immune system [94] and the regulation of glucose metabolism in liver [95].

## CONCLUSIONS

The present study provides evidence for a mechanism of diclofenac induced liver injury that involves pro-inflammatory cytokine and acute phase responses. We propose a molecular circuit that lead to an imbalance of pro- and anti-inflammatory signalling as a cause of what is supposed to be an idiosyncratic liver injury. The methodology employed in the present study may also help to understand immune allergic liver injury induced by other NSAIDS.

## METHODS

### Animals and drug treatment

All animal work followed strictly the Public Health Service (PHS) Policy on Humane Care and Use of Laboratory Animals of the National Institutes of Health, USA. Formal approval to carry out animal studies was granted by the animal welfare ethics committee of Institutional Animal care and Use Committee (IACUC).

C57BL6- mice (males, 8 weeks old) were purchased from the Orient-Bio Co. (Seongnam, Korea). Mice were maintained under laboratory conditions of controlled temperature (23 ± 3°C) and humidity (55 ± 10%) with a 12/12 h light/dark cycle and were given standard food pellets and water *ad libitum*. The sodium salt of diclofenac was purchased from (Sigma-Aldrich, St. Louis, MO) and diluted in sterile saline (Sigma-Aldrich) and administered daily by intraperitoneal injection of 30 mg/kg (low dose, *N* = 6) or 150 mg/kg (high dose, *N* = 6) for up to 14 days. Because of the high mortality observed at the 150 mg/kg dose the genomic studies were performed with tissue samples of the lower dose only. Control mice (*N* = 6) were administered corresponding quantities of saline. The mice were killed at 24 h (day 1), 72 h (day 3) or 14 days after vehicle or diclofenac administration. Furthermore, studies with primary mouse collagen sandwiched hepatocytes were performed using a protocol previously described [96, 97] with the following modifications: Briefly, the portal vein of *N* = 3 mice was cannulated with a 22-gauge plastic cannula and the liver was perfused with a Krebs Ringer buffer (pH 7.4) containing glucose (10 mM) and Hepes (10 mM), at a flow rate of 4 ml/min. The liver was subsequently perfused with a collagenase solution (Calcium containing Krebs Ringer Buffer with glucose) for 10–15 min. Hepatocytes were isolated by removing the capsule and filtering through the cell strainer. Isolated hepatocytes were washed three times with ice cold DMEM containing 10% FBS and hepatocytes of > 90% viability were obtained by using the Optiprep™ density gradient medium (Sigma-Aldrich, St. Louis, MO). The primary hepatocytes were suspended in DMEM medium (Lonza, Verviers, Belgium) and plated on collagen coated dishes. After four hours the medium was discarded and overlaid with a sandwich matrix (1.25 μg/cm^2^ of rat tail collagen). The medium was replaced with fresh HepatoZYME-SFM (Life technologies) medium and after 24 hours of culture primary hepatocytes were treated with 500 μM diclofenac for a further 24 h. The control group was treated with the vehicle only (0.5% v/v DMSO).

### Blood biochemistry

Blood was drawn from the inferior vena cava, and serum was obtained by centrifugation at 3,000 rpm for 30 min at room temperature. Serum aspartate aminotransferase (AST), alanine aminotransferase (ALT), alkaline phosphatase (ALP) and total bilirubin (TBIL) were measured using a Dry Chem-3000 autoanalyzer (FujiFilm, Tokyo, Japan).

### RNA extraction

The liver was explanted from the diclofenac (30 mg/kg) and saline-treated mice, washed free of blood and snap-frozen in liquid nitrogen. Liver samples were subsequently stored in a deep freezer until RNA extraction. Frozen liver samples were immediately added to the RLT buffer containing β-mercaptoethanol and homogenized using a TissueLyser (Qiagen, Hilden, Germany). Total RNA from each tissue was isolated and purified using the RNase mini kit (Qiagen) according to the manufacturer's recommendation. The concentration of total RNA was assessed using a NanoDrop spectrophotometer (NanoDrop Technologies, Wilmington, DE), and RNA integrity was determined with a 2100 Bioanalyzer (Agilent Technologies, Santa Clara, CA).

### Microarray analysis and data capture

Microarray studies were performed with an initial amount of 250 ng total RNA as previously described [98]. All steps of cDNA synthesis, biotin labeling, fragmentation, hybridization, staining, washing and scanning were performed according to the manufacturer's recommendations (Affymetrix, Santa Clara, CA). The Affymetrix GeneChip Mouse 430 2.0 was used for microarray analysis and was scanned using a GeneChip Scanner 3000 (Affymetrix). The microarray scanned image and intensity files (.cel file) were imported into the GenPlex gene expression analysis software (http://genplex.co.kr). Data normalization was performed using MAS 5.0 and Global Median normalization. Differentially expressed genes were selected using a volcano plot based on > 1.5-fold change using Welch's *t*-test (*P* < 0.05) and DEG filtering.

The microarray data were uploaded to the Gene Expression Omnibus (GEO) database [accession number GSE 75277].

### Quantitative real-time RT-PCR analysis

The differentially expressed genes were validated by quantitative real-time RT-PCR. The primers were purchased from GenoTech (Daejeon, Korea). Total RNA (2 μg) was reverse-transcribed with SuperScript II (Invitrogen, Carlsbad, CA) using an oligo-dT primer as described by the manufacturer. cDNA samples were stored at −20°C until use. Quantitative real-time RT-PCR was performed in a 20 μl reaction volume containing 0.5 μl (10pM) forward and reverse specific primers, 10 μl of SYBR Green master mix (Applied Biosystems, Carlsbad, Califomia), 2 μl of cDNA and 7 μl of nuclease-free water. The cDNA was amplified using a StepOne and StepOnePlus Real-Time PCR System (Applied Biosystems) following the manufacturer's protocol. The *18S* ribosomal RNA primers were used as an internal control. Note, the primer sequences of all genes investigated are listed in Table S1.

### Western blot analysis

Liver tissue from diclofenac (30 mg/kg) and saline-treated mice (day 1, 3 and 14) was removed, snap-frozen in liquid nitrogen and kept in a deep freezer until further processing. The frozen tissue together with 600 μl of PRO-PREPTM solution (iNtRON, Seongnam, Korea) was homogenized with a TissueLyser (Qiagen, Hilden, Germany); the homogenate was incubated on ice for 30 min and centrifuged at 13,000 rpm and 4°C for 5 min. The supernatant was transferred to a new tube and the protein concentration was determined with the Bradford Protein Assay (Bio-Rad, Hercules, CA). Subsequently, the samples were heated with Bolt^™^ sampling buffer (Life technologies, Grand Island, NY) and reducing agent (Invitrogen, Waltham, MA) at 70°C for 10 min. An equal amount of protein lysates was loaded to Bolt™ bio-tris plus mini gels (Life technologies, Grand Island, NY) and blotted onto a nitrocellulose membrane (Millipore, Bedford, MA). After protein transfer, membranes were blocked with Tris-buffered saline (TBS)/0.1% Tween 20 (ScyTek, West Logan, UT) and 5% (w/v) nonfat dry milk for 1 h at room temperature, and thereafter probed with the indicated primary antibodies (1:200~1:1000) overnight at 4°C. All antibodies were diluted with SuperBlock T20 (PBS) blocking buffer (Thermo, Waltham, MA). After washing of the membranes these were incubated with horseradish peroxidase (HRP)-conjugated secondary antibodies (1:2000) for 2 h. Once again the membranes were washed to remove excess secondary antibody and bands were visualized using the enhanced chemiluminescence (ECL) detection system (Pierce Biotechnology, Rockford, IL) according to the manufacturer's recommendations. The anti-STAT3, anti-phosphorus STAT3, anti-fibronectin, anti-M-CSF, anti-RC-PTP, anti-actin antibodies and secondary antibody were purchased from Santa Cruz Biotechnology (Santa Cruz, CA). The anti-GHR was purchased from Abcam (Cambridge, UK).

List of antibodies used in the western blot:

**Table:** 

Antibody	Vendor	Cat no.	Lot number
**Growth hormone receptor**	Abcam	ab134078	YI05181 0CS
**CTLA4**	Abcam	ab134090	
**Actin (C-11)**	Santa Cruz Biotechnology	sc-1615	E0913
**APCS**	Santa Cruz Biotechnology	sc69796	SAP(6E6)
**Fibronectin (EP5)**	Santa Cruz Biotechnology	sc-8422	G1812
**M-CSF (D-4)**	Santa Cruz Biotechnology	sc-365779	A0912
**p-STAT3 (B-7)**	Santa Cruz Biotechnology	sc-8029	J0813
**STAT3 (C-20)**	Santa Cruz Biotechnology	sc-482	I1113
**TC-PTP (S-17)**	Santa Cruz Biotechnology	sc-102129	
**CD44**	R & D SYSTEMS	AF6127	

### Histopathology

Using standard operating procedures of the laboratory a range of stains were employed to evaluate the liver morphology of control and diclofenac treated mice and included Hematoxylin and eosin (H & E), Periodic acid-Schiff reaction (PAS), PAS diastase digestion, Elastica van Gieson, silver and Prussian blue.

### Immunohistochemistry

Livers from control and diclofenac treated animals were fixed in 4% buffered paraformaldehyde and embedded in paraffin block using standard protocols of the laboratory. 1 μm thick sections were deparaffinised and rehydrated through a descending alcohol series followed by a 4 min washing step in distilled H2O. Subsequently, antigen retrieval was performed in citrate buffer (pH 6) in a water bath at 98°C for 30 min. The ZytoChem-Plus HRP Polymer-Kit of Zytomed Systems, Germany was used for immunohistochemistry. The slides were rinsed with distilled H2O and after a 5 min incubation step in tris-buffered saline (washing buffer), endogenous peroxidase activity was blocked with 3% peroxidase blocking reagent (Merck, Germany) for 5 min followed by a second washing step. Thereafter, the sections were blocked for 5 min with protein-block serum free reagent (ZytoChem-Plus HRP Polymer-Kit, reagent 1) and incubated with primary antibodies for 60 min. The antibodies were purchased from diverse vendors and diluted with washing buffer as given in parenthesis: CD 31 (DAKO clone JC 70 A, Germany diluted to 1:75), CD68 (DAKO clone KP-1, Germany diluted to 1:100), Lipopolysacharide binding protein sc-14666 (Santa Cruz Biotechnology, Inc., USA diluted to 1: 25), Tc-PTP sc-102192 (Santa Cruz Biotechnology, Inc., USA diluted to 1:75), macrophage colony stimulating factor sc-365779 (Santa Cruz Biotechnology, Inc., USA diluted to 1:300), Ki-67 (Thermo Fischer clone SP 6, Germany diluted to 1:100) fibronectin sc-8442 (Santa Cruz Biotechnology, Inc., USA diluted to 1:50), growth hormone receptor ab134087 (Abcam, UK diluted to 1:300), Leptin receptor sc-18931 (Santa Cruz Biotechnology, Inc., USA diluted to 1:50) and Hep Par1 clone OCH1E5 (Santa Cruz Biotechnology sc-58693, Inc., USA diluted to 1:800). The bound primary antibodies or bridging antibodies were incubated with labelled polymer HRP Anti-Rabbit or anti Mouse secondary antibody (ZytoChem-Plus HRP Polymer-Kit, reagent 2) for 20 min. Subsequently, the reaction was developed and visualized by use of reagent 3 of the ZytoChem-Plus HRP Polymer-Kit and by placing the slides in a moist chamber at room temperature allowing an incubation time of 30 min.

Finally, the sections were counterstained with Haematoxylin for 5 min, washed under running warm tap water for 10 min and dehydrated in a cabinet at 60°C for 20 min, coverslipped and examined under a light microscope (Nikon Ni-E microscope, Japan). Image capture and photo-documentation was done with the Nikon NIS basic research microscopic imaging software version 4.3.

The images were converted into Tiff files and unless otherwise stated the images were finalised in Adobe Photoshop version CS5. With the exception of Ki67 and Hep Par 1 all immunohistochemistry images are shown in the auto-colour function mode.

### Primary genomic data analysis

Commonly expressed genes amongst different treatment groups were determined by employing a Venn diagram analysis, and inflammatory, immune and stress response genes were sorted by biological process and gene ontology using the GenPlex software version 3.0. The functions and canonical pathways of the differentially expressed genes were subsequently interrogated using the Ingenuity Pathway Analysis (IPA) software version 9.0; (Ingenuity Systems, Redwood City, CA). This system is continuously updated with new scientific publications, review articles, text books and KEGG ligand information stored in the ingenuity system knowledge base. *P*-values < 0.05 were considered as statistically significant and therefore non-random association. *P*-values were calculated using the right-tailed Fisher exact test.

### Bioinformatic analysis

The microarray expression data were normalized using the MAS5 algorithm available within the GeneXplain 3.0 platform (http://www.genexplain.com/). Then, differentially expressed genes (DEGs) were determined by applying a hypergeometric test to the normalised data sets with a cut-off ≥ ± 1.5 fold change and *p*-value < 0.01. Genes satisfying these conditions were grouped as up- and down-regulated and a heat map for DEGs was created based on the average-linkage hierarchical clustering with Euclidean distance (ArrayTrack software Version 3.5.0 [99]).

### Functional enrichment analysis

DEGs were mapped to various ontologies, i.e. biological processes, cellular components, molecular functions, reactome pathways and transcription factor classification. For each ontological term respective *p*-values were calculated using the GeneXplain platform, Version 3.0.

### Gene regulatory network analysis

Regulatory gene networks were constructed with an analysis tool of the GeneWays database [http://wiki.biouml.org/index.php/Geneways] available within the GeneXplain platform Version 3.0. A default cut-off score of 0.2, a FDR < 0.05 and a Z-score at 1.0 with a maximum radius of 4 steps upstream of an input gene set was set to identify statistically significant master regulatory genes. Note, the GeneWays tool is an integrated system used to automatically extract, analyse, visualize and integrate molecular pathway data from published peer reviewed articles and more than eight million abstracts [100].

### Transcription factor binding site analysis

The TRANSFAC^®^ 2013.2 library of positional weight matrices and ‘vertebrate_non_redundant_minSUM’ profile was used for an identification of transcription factor binding sites (TFBS) in promoters of up- and down-regulated genes (Yes-set). Specifically, promoters of DEGs were extracted using an automated procedure with respect to the transcriptional start site (TSS) and contained the TFIIB recognition element and the TATA-box at the 5′-end, an initiator region around the TSS and a downstream promoter element (DPE) at the 3′-end [101]. The extracted promoters were interrogated for cis-regulatory binding sites with a length of −1000 to +100 base pairs relative to the TSS. Overrepresentation of transcription factor binding sites in promoters of differentially expressed genes were identified by comparing individual sites in promoters of DEG (= Yes-set) and non-regulated genes (= No-set) as previously described by Stegmaier et al. 2011 [102].

### Construction of composite modules

A composite module is a particular combination of positional weight matrices (PWM) for different transcription factors and is based on an analysis of individual TFBS with a test for pairwise co-occurrences of binding sites. As described recently, the approach represents a variant of the F-MATCH algorithm for binding site pairs and quantifies overrepresentation of promoter sequences with sites of both PWMs in the foreground set using the Fisher test [102–104]. Specifically, DEGs implicated in the inflammation, immune and stress response were considered for composite module (CM) construction, and the procedure is based on the primary multi-component fitness function as to ascertain co-occupancy of different transcription factors in promoters of regulated genes [102, 104]. The default filtering criteria of the GeneXplain platform was used and statistically significant composite modules were determined.

## SUPPLEMENTARY MATERIALS FIGURES AND TABLES

















## Abbreviations

NSAID: Nonsteroidal anti-inflammatory drug
DILI: Drug-induced liver injury
ALT: Alanine aminotransferase
AST: Serum aspartate aminotransferase
ALP: Alkaline phosphatase
TBIL: Total bilirubin
H & E: Hematoxylin and eosin
PAS: Periodic acid-Schiff
LPS: Lipopolysaccharide
Th-17: T helper 17 cells
DEGs: Differentially expressed genes
IPA: Ingenuity Pathway Analysis
TFBS: Transcription factor binding sites
TSS: Transcription start site
PWM: Position weight matrix
DPE: Downstream promoter element
CMs: Composite modules
PPI: Protein-protein interaction
ROS: Reactive oxygen species
