# Supplementary material for: Immunogenomics reveal molecular circuits of diclofenac induced liver injury in mice
Source: Oncotarget. 2016 Feb 25;7(12):14983–5017. doi: 10.18632/oncotarget.7698 (PMC4924767; doi:10.18632/oncotarget.7698)
Supplement: Supplementary file 1 [file oncotarget-07-14983-s001.pdf]

Immunogenomics reveal molecular circuits of diclofenac induced liver injury in mice

Supplementary Materials

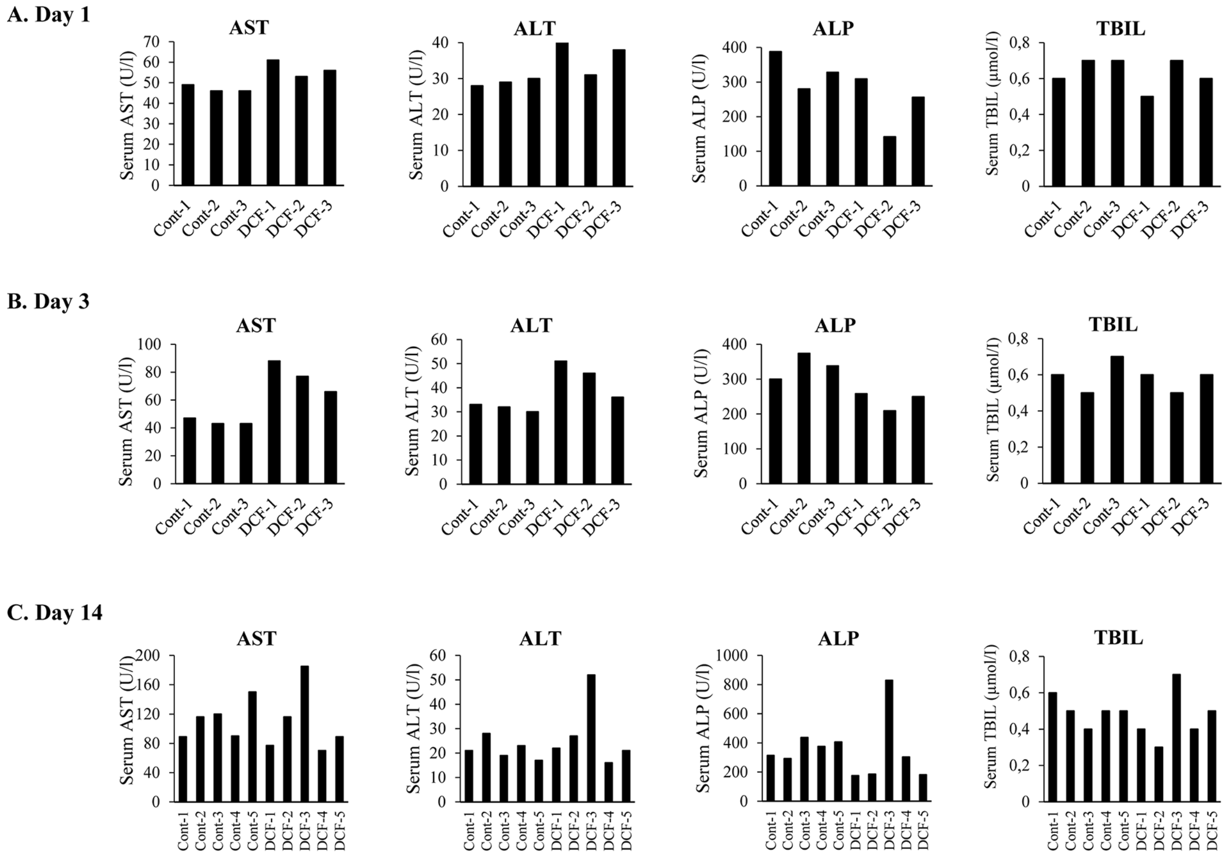

Supplementary Figure S1: Individual serum AST, ALT, ALP and TBIL values of diclofenac-treated mice. Diclofenac was given to mice for day 1, 3 and 14.

A B C

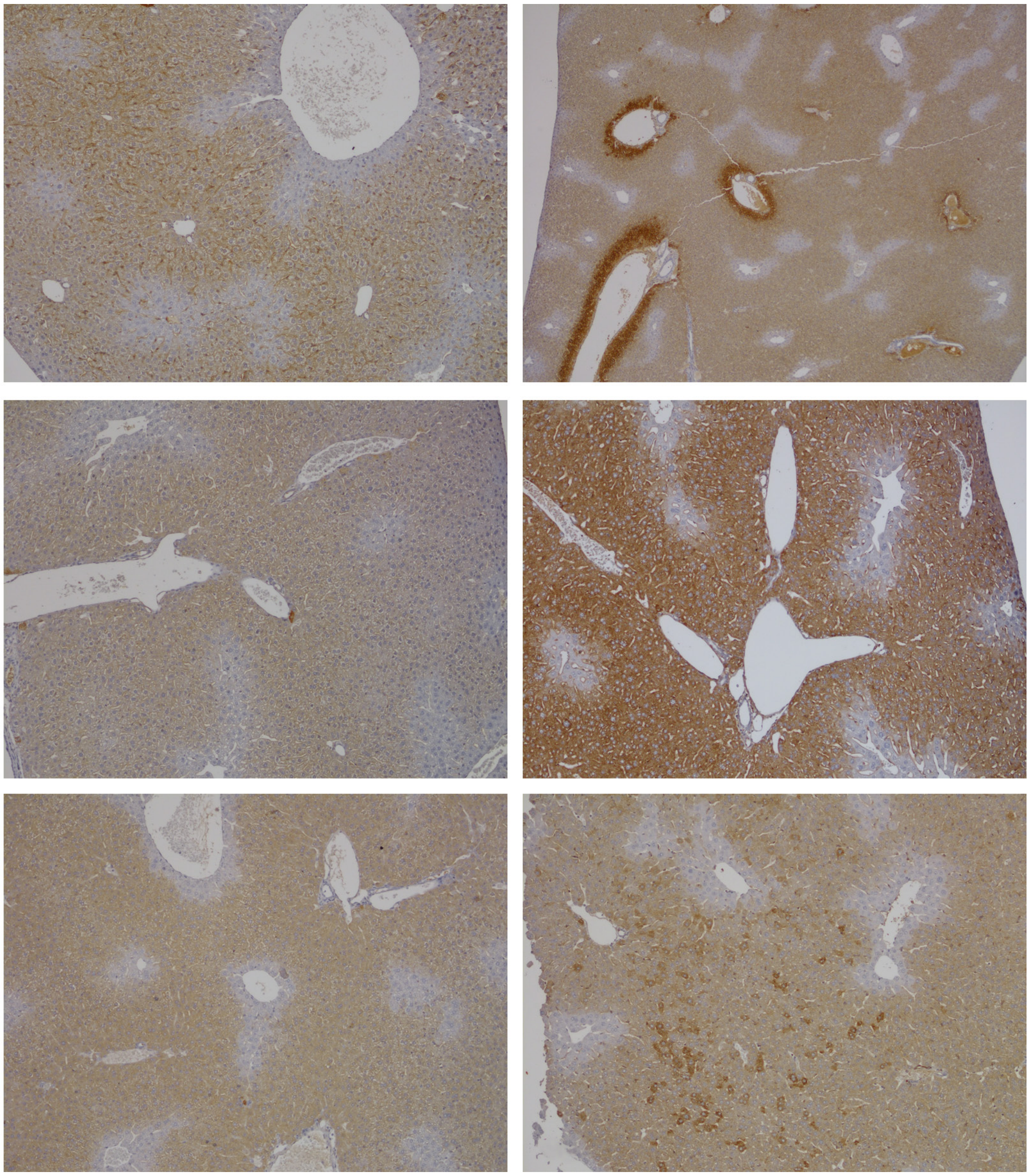

Supplementary Figure S2: Immunohistochemistry of Hep Par 1 in livers of control and diclofenac treated mice. (**A**) from the left to the right: Control and repeated dose 30 mg/kg diclofenac (day 3). (**B**) from the left to the right: Control and repeated dose 30 mg/kg diclofenac (day 14). (**C**) from the left to the right: Control and repeated dose 150 mg/kg diclofenac (day 3). Magnifications 70x.

A

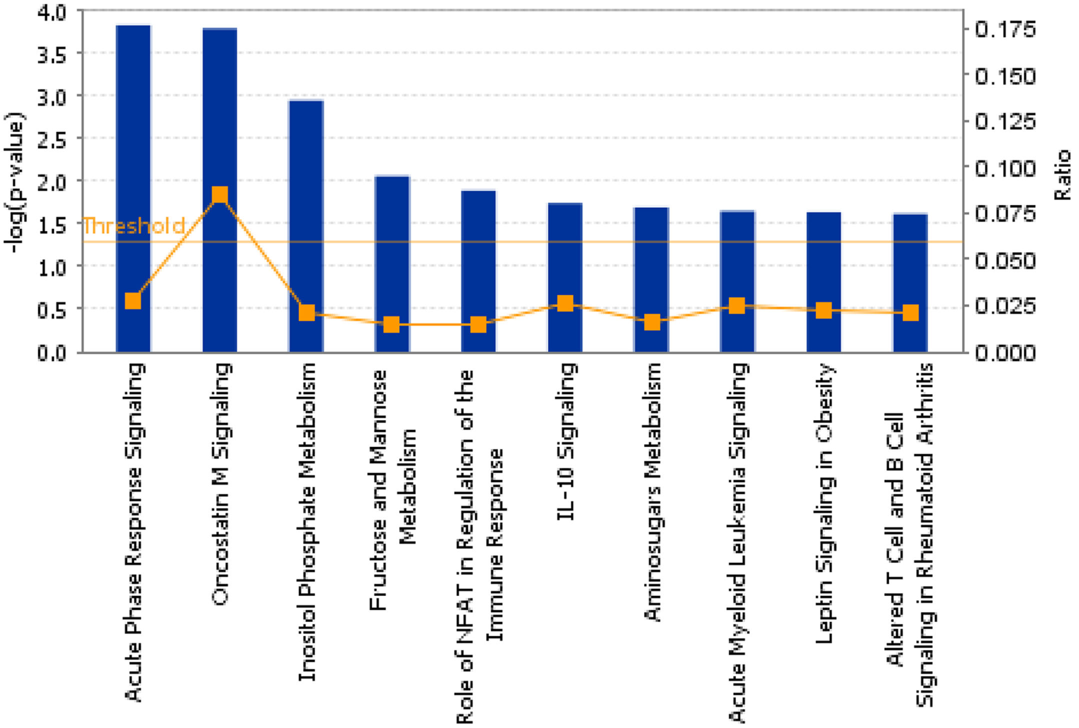

B

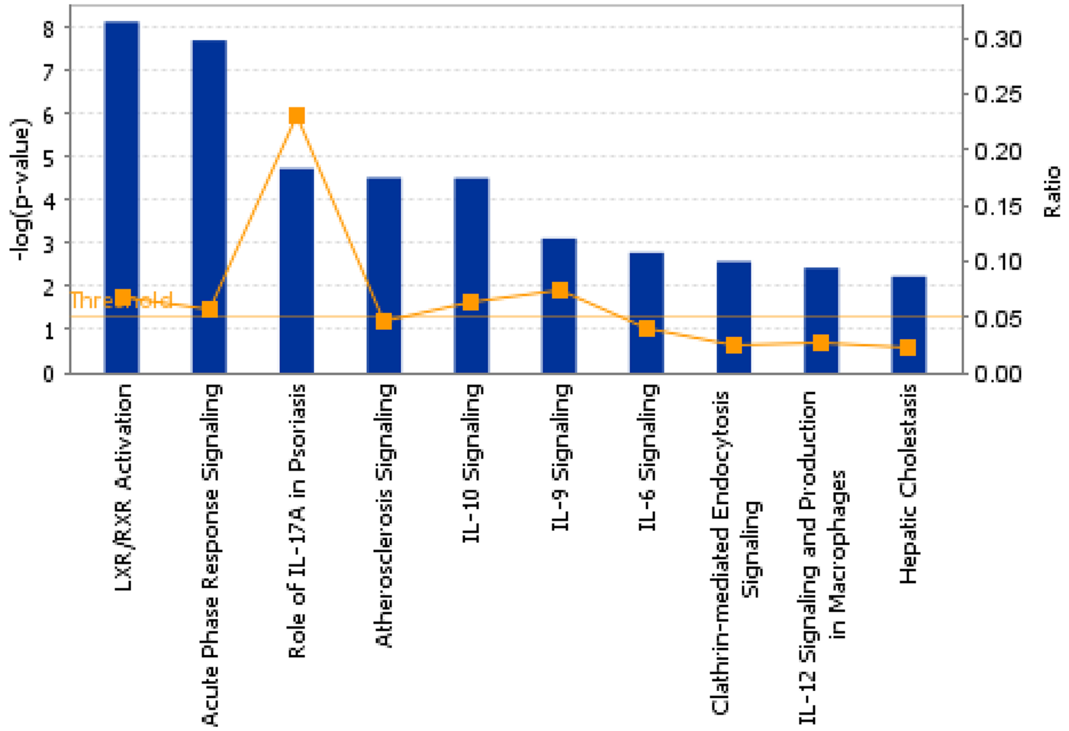

Supplementary Figure S3: Top 10 canonical pathways after single and repeated diclofenac treatment of mice. Genes were selected based on a > 1.5 – fold change ($P < 0.05$) cutoff using the GenPlex program. The canonical pathways were analysed using the Ingenuity Pathway Analysis program.

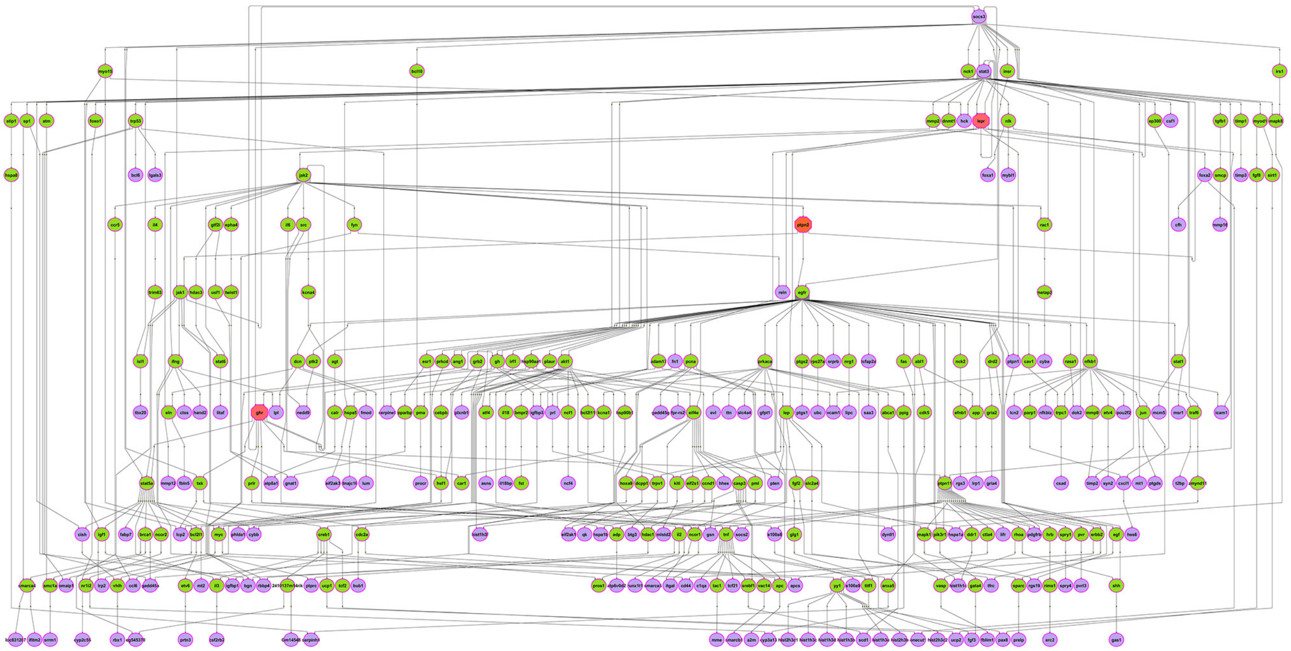

Supplementary Figure S4: Integrated master regulatory network after single diclofenac treatment of mice. Based on database information 3 different master regulatory networks were fused using the GeneXplain software. The color coding red, blue and green represent nodes for master regulator, regulated genes and connecting genes, respectively. The network consisted of 146 DEGs of 304 interacting partners.

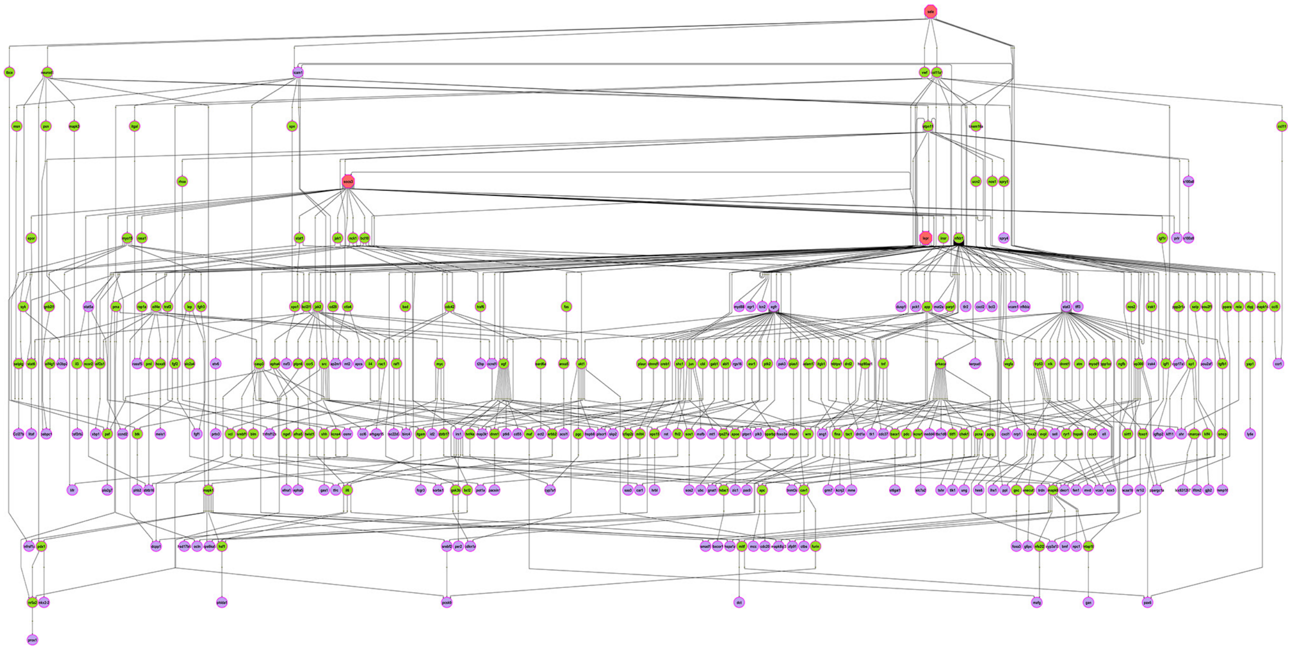

Supplementary Figure S5: Integrated master regulatory network after repeated diclofenac treatment of mice for 3 days. Based on database information 3 different master regulatory networks were fused using the GeneXplain software. The color coding red, blue and green represent nodes for master regulator, regulated genes and connecting genes, respectively. The network consisted of 159 DEGs of 330 interacting partners.

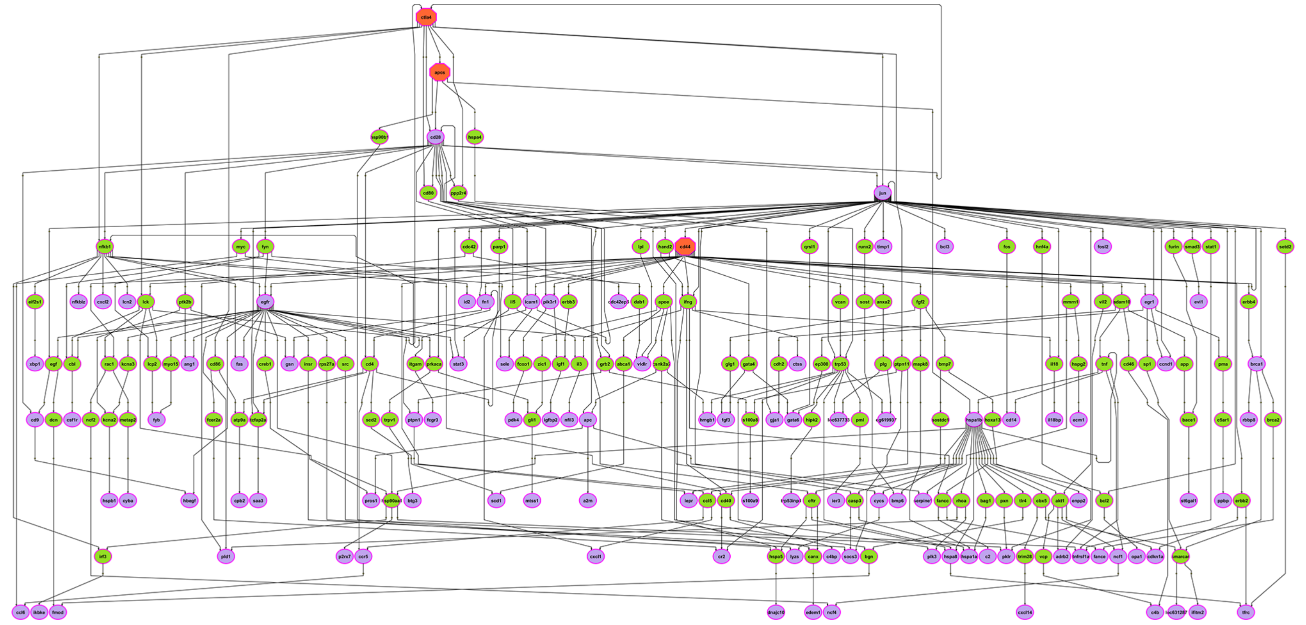

Supplementary Figure S6: Integrated master regulatory network after repeated diclofenac treatment of mice for 14 days. Based on database information 3 different master regulatory network were fused using the GeneXplain software. The color coding red, blue and green represent nodes for master regulator, regulated genes and connecting genes, respectively. The network consisted of 97 DEGs of 207 interacting partners.

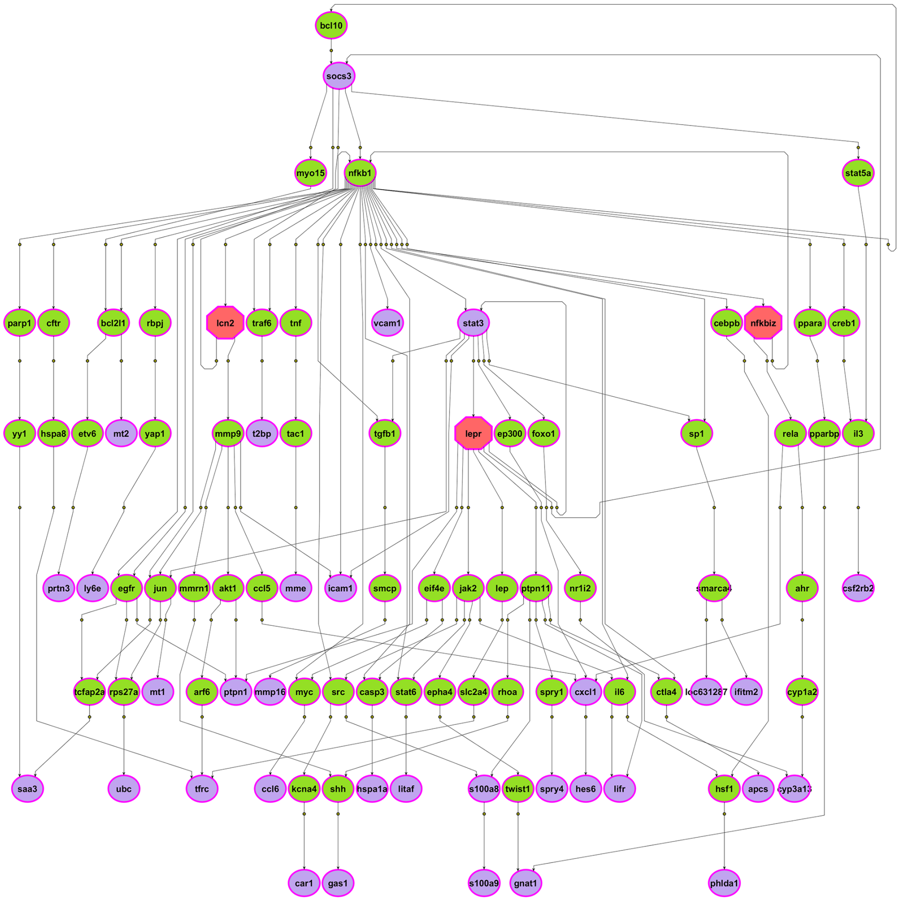

Supplementary Figure S7: Integrated master regulatory network for genes commonly regulated after single and repeated treatment of mice for 3 days. Based on database information 3 different master regulatory network were fused using the GeneXplain software. The color coding red, blue and green represent nodes for master regulator, regulated genes and connecting genes, respectively. The network consisted of 36 DEGs of 93 interacting partners.

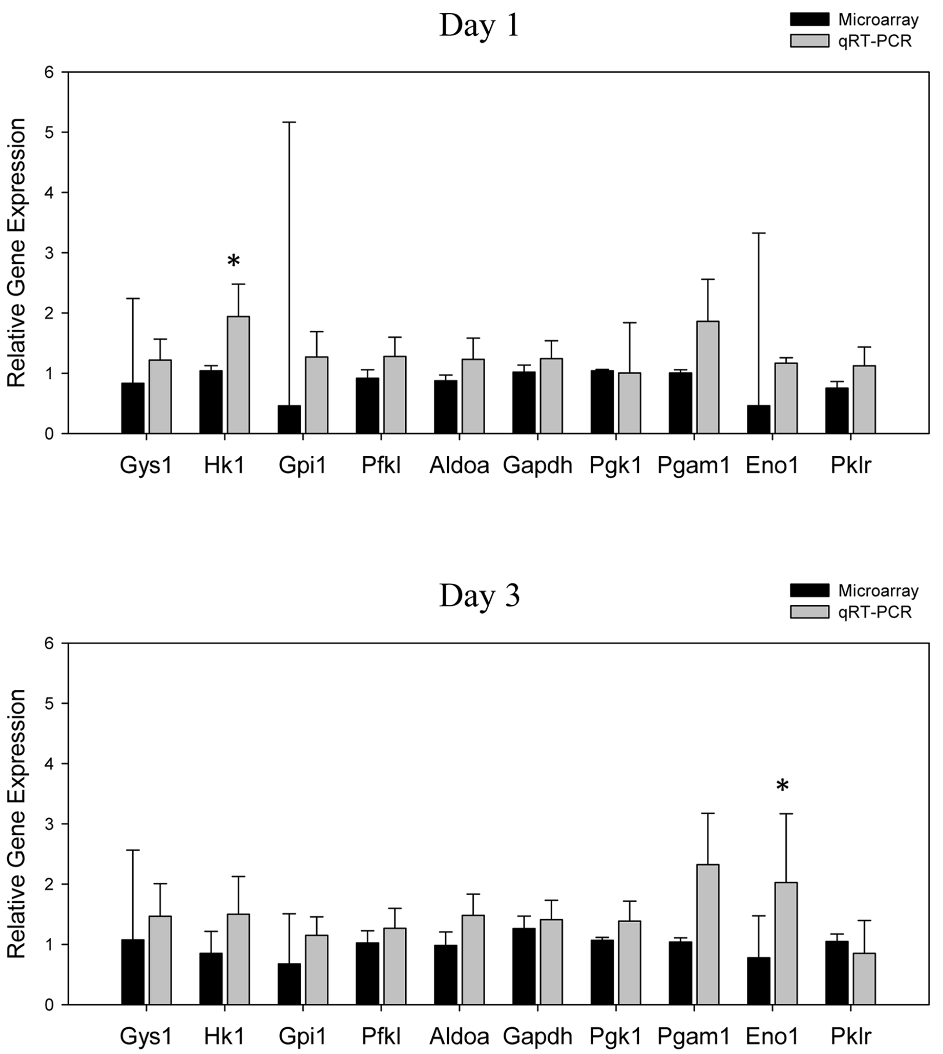

Supplementary Figure S8: qRT-PCR of of genes involved in glycogenesis and carbohydrate metabolism. The *y*-axis indicates the relative fold change in expression (diclofenac-treated *vs*. controls). Data are means ± SD ($n = 3$). *$P < 0.05$, **$P < 0.01$.

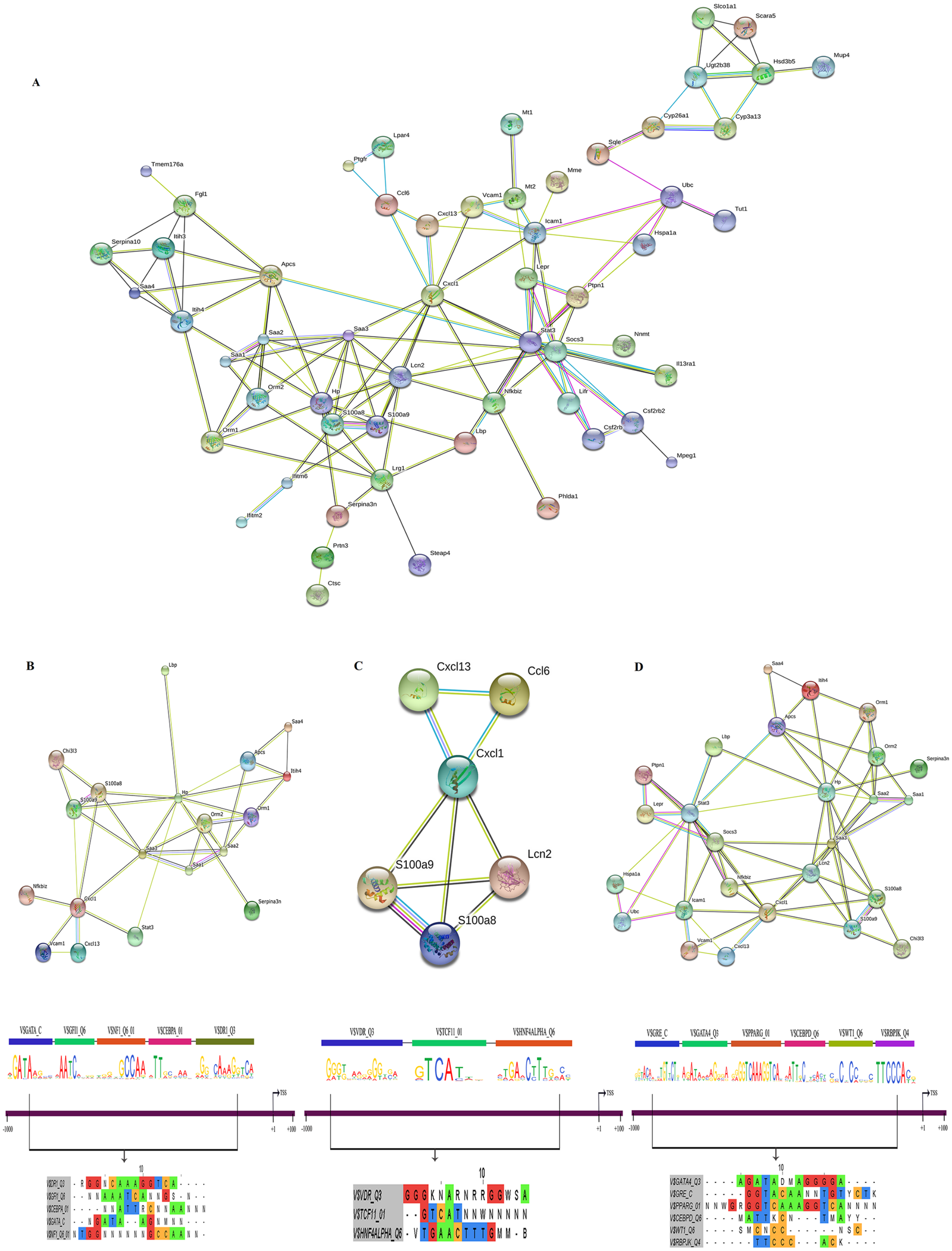

Supplementary Figure S9: Protein interaction network of commonly regulated genes after single (day 1) and repeated diclofenac treatment (day 3). (**A**) Protein interaction network of entire DEGs. (**B**) Inflammatory response sub-network with its composite module of co-bound transcription factors at promoters of regulated genes. (**C**) Immune response sub-network with its composite module of co-bound transcription factors at promoters of regulated genes. (**D**) Stress response sub-network with its composite module of co-bound transcription factors at promoters of regulated genes.

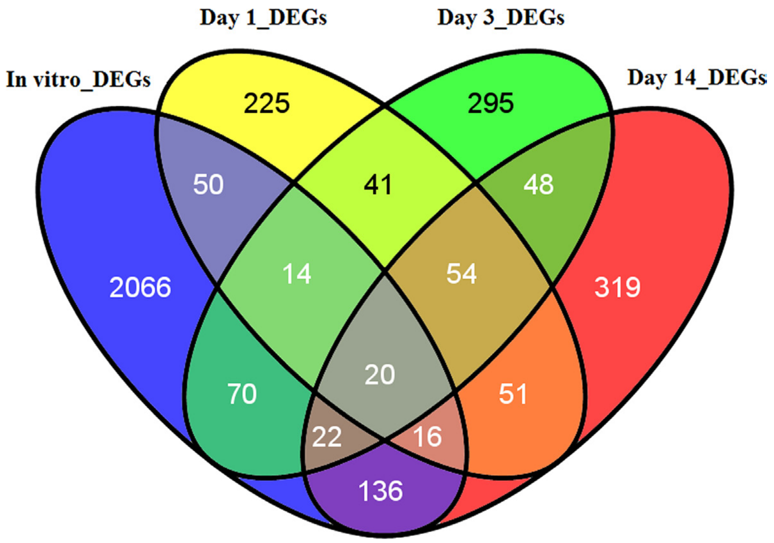

Supplementary Figure S10: Venn diagram of DEGs obtained from *in vitro* and *in vivo* diclofenac treatment. Twenty genes were commonly regulated in both *in vitro* and *in vivo* diclofenac treatment conditions and are given in Table S16.

Supplementary Table S1: Primer sequences used in real-time RT-PCR analyses

Target	Primer	Primer Sequence	Product Size (base pair)
mStat3	Forward	5′-TGCAGAGCAGGTATCTTGAG-3′	157
	Reverse	5′-TGCTGCTTCTCTGTCACTAC-3′	
mLbp	Forward	5′-CCGGCATCCGGTTGAACACCA-3′	202
	Reverse	5′-AAGACAGGTTCCCGGGCGGA-3′	
mFas	Forward	5′-CCGAATGTCGCAGAACCTTA-3′	150
	Reverse	5′-GGCAGGCTCTCTCCTCTCTT-3′	
mPtprj	Forward	5′-GAACACCGTGGACGTGTATG-3′	180
	Reverse	5′-GTCATTGCCGTTGTGTTCTG-3′	
mIL-13ra1	Forward	5′-CTGACAGTCTTGCAGCATGG-3′	184
	Reverse	5′-CCTACAAGTTTCCAATAAGCAATG-3′	
mMyd88	Forward	5′-CTCCCCTGGGCTCAACCCGT-3′	204
	Reverse	5′-TGGCCAGGCCCCCTTGATGA-3′	
mGstm3	Forward	5′-TCATTGGTGCCCAGACCCAACC-3′	225
	Reverse	5′-GCCTGGGGCAGCTCCTTAAAC-3′	
mRor-α	Forward	5′-GGACATTAGCTGGCTGCTTC-3′	162
	Reverse	5′-CCTTGTCTGGGGTTTGTCAT-3′	
hmr_18S rRNA	Forward	5′-GTAACCCGTTGAACCCCATT-3′	151
	Reverse	5′-CCATCCAATCGGTAGTAGCG-3′	

Supplementary Table S2: Lists of genes involved in immune, inflammatory and stress responses in diclofenac-treated mice liver. The differentially expressed genes were selected from the livers of mice treated with diclofenac for 1, 3 and 14 days. Genes were sorted by biological process and gene ontology using the GenePlex and GeneXplain software.

Supplementary Table S3: Differentially expressed genes coding for drug metabolizing enzymes in liver after single and repeated diclofenac treatment (30 mg/kg, *i.p.)*

1 DAY			
**Probe ID**	**Gene_symbol**	**Description**	**Average Fold Change**
**Phase I**			
1419430_at	Cyp26a1	Cytochrome P450, family 26, polypeptide 1, subfamily a	−2.23
1422230_s_at	Cyp2a5	Cytochrome P450, family 2, polypeptide 5, subfamily a	−1.45
1419590_at	Cyp2b9	Cytochrome P450, family 2, polypeptide 9, subfamily b	1.89
1423244_at	Cyp2c40	Cytochrome P450, family 2, polypeptide 40, subfamily c	−1.58
1419582_at	Cyp2c55	Cytochrome P450, family 2, polypeptide 55, subfamily c	−1.67
1423244_at	Cyp2c68	Cytochrome P450, family 2, polypeptide 68, subfamily c	−1.58
1426102_at	Cyp2j13	Cytochrome P450, family 2, polypeptide 13, subfamily j	2.07
1419523_at	Cyp3a13	Cytochrome P450, family 3, polypeptide 13, subfamily a	1.75
**Phase III**			
1456586_x_at	Mvp	Major vault protein	1.44
1438377_x_at	Slc13a3	Solute carrier family 13 (sodium-dependent dicarboxylate transporter), member 3	2.48
1434473_at	Slc16a5	Solute carrier family 16 (monocarboxylic acid transporters), member 5	1.56
1428379_at	Slc17a6	Solute carrier family 17 (sodium-dependent inorganic phosphate cotransporter), member 6	1.45
1449215_at	Slc22a21	Solute carrier family 22 (organic cation transporter), member 21	1.47
1419751_x_at	Slc22a27	Solute carrier family 22, member 27	1.45
1420835_at	Slc25a30	Solute carrier family 25, member 30	−1.75
1449067_at	Slc2a2	Solute carrier family 2 (facilitated glucose transporter), member 2	−1.49
1422497_at	Slc30a5	Solute carrier family 30 (zinc transporter), member 5	1.52
1448741_at	Slc3a1	Solute carrier family 3, member 1	2.60
1452445_at	Slc41a2	Solute carrier family 41, member 2	3.58
1441047_at	Slc45a4	Solute carrier family 45, member 4	−1.89
1456297_at	Slc46a2	Solute carrier family 46, member 2	−1.42
1434096_at	Slc4a4	Solute carrier family 4 (anion exchanger), member 4	1.54

1417929_at	Slc7a8	Solute carrier family 7 (cationic amino acid transporter, y+ system), member 8	1.67
1420379_at	Slco1a1	Solute carrier organic anion transporter family, member 1a1	−1.78
1420405_at	Slco1a4	Solute carrier organic anion transporter family, member 1a4	−1.49

3 DAY

Probe ID	Gene_symbol	Description	Average Fold Change
**Phase I**			
1451260_at	Aldh1b1	Aldehyde dehydrogenase 1 family, member B1	1.56
1436119_at	Aldh1l2	Aldehyde dehydrogenase 1 family, member L2	−1.62
1435371_x_at	Ces3	Carboxylesterase 3	−2.55
1417017_at	Cyp17a1	Cytochrome P450, family 17, polypeptide 1, subfamily a	1.83
1419430_at	Cyp26a1	Cytochrome P450, family 26, polypeptide 1, subfamily a	−2.71
1421363_at	Cyp2c39	Cytochrome P450, family 2, polypeptide 39, subfamily c	1.45
1418780_at	Cyp39a1	Cytochrome P450, family 39, polypeptide 1, subfamily a	1.52
1419523_at	Cyp3a13	Cytochrome P450, family 3, subfamily a, polypeptide 13	1.57
1426064_at	Cyp3a44	Cytochrome P450, family 3, polypeptide 44, subfamily a	1.47
1424853_s_at	Cyp4a10	Cytochrome P450, family 4, polypeptide 10, subfamily a	−1.54
1440134_at	Cyp4a31	Cytochrome P450, family 4, subfamily a, polypeptide 31	−1.56
1422533_at	Cyp51	Cytochrome P450, family 51	1.69
1450646_at	Cyp51	Cytochrome P450, family 51	1.69
1438743_at	Cyp7a1	Cytochrome P450, family 7, subfamily a, polypeptide 1	2.03
**Phase II**			
1427474_s_at	Gstm3	Glutathione S-transferase, mu 3	1.87
1432517_a_at	Nnmt	Nicotinamide N-methyltransferase	1.80
**Phase III**			
1450245_at	Slc10a2	Solute carrier family 10, member 2	2.93
1417600_at	Slc15a2	Solute carrier family 15 (h+/peptide transporter), member 2	−1.62
1434473_at	Slc16a5	Solute carrier family 16 (monocarboxylic acid transporters), member 5	1.53

1456003_a_at	Slc1a4	Solute carrier family 1 (glutamate/neutral amino acid transporter), member 4	1.52
1436162_at	Slc22a28	Solute carrier family 22, member 28	−1.49
1450404_at	Slc23a1	Solute carrier family 23 (nucleobase transporters), member 1	1.42
1450018_s_at	Slc25a30	Solute carrier family 25, member 30	−2.10
1424211_at	Slc25a33	Solute carrier family 25, member 33	−1.82
1422497_at	Slc30a5	Solute carrier family 30 (zinc transporter), member 5	1.75
1455249_at	Slc36a4	Solute carrier family 36 (proton/amino acid symporter), member 4	1.56
1426601_at	Slc37a1	Solute carrier family 37 (glycerol-3-phosphate transporter), member 1	1.96
1427035_at	Slc39a14	Solute carrier family 39 (zinc transporter), member 14	2.00
1448741_at	Slc3a1	Solute carrier family 3, member 1	2.23
1460565_at	Slc41a1	Solute carrier family 41, member 1	1.97
1452445_at	Slc41a2	Solute carrier family 41, member 2	2.75
1453255_at	Slc43a1	Solute carrier family 43, member 1	1.45
1417022_at	Slc7a3	Solute carrier family 7 (cationic amino acid transporter, y+ system), member 3	1.79
1426068_at	Slc7a4	Solute carrier family 7 (cationic amino acid transporter, y+ system), member 4	1.58
1449844_at	Slco1a1	Solute carrier organic anion transporter family, member 1a1	−1.77
1420379_at	Slco1a1	Solute carrier organic anion transporter family, member 1a1	−1.77
1420405_at	Slco1a4	Solute carrier organic anion transporter family, member 1a4	−1.42

14 DAY

Probe ID	Gene_symbol	Description	Average Fold Change
**Phase I**			
1424576_s_at	Cyp2c44	Cytochrome P450, family 2, polypeptide 44, subfamily c	−1.44
1424273_at, 1440327_at	Cyp2c70	Cytochrome P450, family 2, polypeptide 70, subfamily c	2.81
1418780_at	Cyp39a1	Cytochrome P450, family 39, polypeptide 1, predicted gene 14336, subfamily a	−1.43
1424352_at	Cyp4a12a	Cytochrome P450, family 4, polypeptide 12a, subfamily a	−1.78
1430172_a_at	Cyp4f16	Cytochrome P450, family 4, polypeptide 16, predicted gene 9705, subfamily f	1.60
1417071_s_at	Cyp4v3	Cytochrome P450, family 4, polypeptide 3, subfamily v	−1.42

1422100_at	Cyp7a1	Cytochrome P450, family 7, polypeptide 1, predicted gene 12704, subfamily a	−1.89
1449309_at	Cyp8b1	Cytochrome P450, family 8, polypeptide 1, subfamily b	−1.84
**Phase II**			
1427474_s_at	Gstm3	Glutathione s-transferase, mu 3	−1.59
**Phase III**			
1428776_at	Slc10a6	Solute carrier family 10 (sodium/bile acid cotransporter family), member 6	6.63
1452653_at	Slc25a22	Solute carrier family 25 (mitochondrial carrier glutamate), member 22	−1.79
1420836_at	Slc25a30	Solute carrier family 25, member 30	−1.64
1427035_at	Slc39a14	Solute carrier family 39 (zinc transporter), member 14	2.99
1448741_at	Slc3a1	Solute carrier family 3, member 1	3.76
1460565_at	Slc41a1	Solute carrier family 41, member 1	1.95
1452445_at	Slc41a2	Solute carrier family 41, member 2	7.26
1416464_at	Slc4a1	Solute carrier family 4 (anion exchanger), member 1	1.46
1427221_at	Slc6a20a	Solute carrier family 6 (neurotransmitter transporter), member 20a	1.42
1427348_at	Slc8a2	Solute carrier family 8 (sodium/calcium exchanger), member 2	1.91
1438115_a_at	Slc9a3r1	Solute carrier family 9 (sodium/hydrogen exchanger), member 3 regulator 1	1.67
1439368_a_at	Slc9a3r2	Solute carrier family 9 (sodium/hydrogen exchanger), member 3 regulator 2	−1.42
1449844_at	Slco1a1	Solute carrier organic anion transporter family, member 1a1	−1.60
1420405_at	Slco1a4	Solute carrier organic anion transporter family, member 1a4	−1.54

Supplementary Table S4: Enriched biological processes after single dose diclofenac treatment

GO ID	Biological Process	No of genes	Genes	*P*-value
GO:0006954	Inflammatory response	35	A2m, Aim2, Apcs, Cd163, Cd44, Chi3l3, Csf1, Cxcl1, Cxcl13, Cyba, Cybb, Fn1, Fpr2, Hck, Hp, Itgal, Itgb2, Itih4, Lbp, Nfkbiz, Orm1, Orm2, Prok2, Reg3b, S100a8, S100a9, Saa1, Saa2, Saa3, Saa4, Serpina3n, Stat3, Tfrc, Themis2, Vcam1	4.7499×10^{-12}
GO:0006955	Immune response	52	Adamdec1, Aim2, Bcl6, C1qa, C1qb, C1qc, Ccl6, Cfh, Clec4n, Csf1, Ctsc, Ctss, Cxcl1, Cxcl13, Cxcl14, Cyba, Cybb, Fcer1g, Fgf3, Hck, Herc6, Ifitm2, Il18bp, Il2rg, Itgb2, Lbp, Lcn2, Lcp2, Lgals3, Lgmn, Lilrb3, March1, Marco, Ncf4, Orm1, Pdgfrb, Pou2f2, Prg4, Procr, Pros1, Prss3, Pten, Raet1d, Raet1e, S100a8, S100a9, Saa1, Trat1, Tyrobp, Ubc, Vsig4, Zbp1	2.7604×10^{-10}
GO:0006950	Response to stress	112	A2m, Aim2, Apcs, Asns, Bcl6, Btg3, C1qa, C1qb, C1qc, Car3, Cd163, Cd44, Cd5l, Cfh, Chi3l3, Clec2h, Clec4n, Col3a1, Cotl1, Csf1, Ctss, Cxcl1, Cxcl13, Cyba, Cybb, Derl1, Dnajb9, Dnajc10, Eif2ak1, Eif2ak3, Fabp5, Fcer1g, Fgf3, Fmod, Fn1, Fpr2, Gabra5, Gadd45a, Gadd45g, Gbp3, Gck, Gfpt1, Gsn, Gsta4, Hamp2, Hck, Herc6, Hhex, Hist2h2be, Hp, Hsd3b2, Hspa1a, Hspa1b, Icam1, Ifitm2, Igfbp1, Il18bp, Inhbb, Itgal, Itgb2, Itih4, Lbp, Lcn2, Lcp2, Lepr, Lgals3, Lgmn, Lilrb3, Lipc, Lpl, Marco, Mnda, Nfkbiz, Nrep, Orm1, Orm2, Pdgfrb, Plac8, Pmaip1, Prl, Prok2, Pros1, Prss3, Pten, Ptgs1, Ptpn1, Ptprc, Raet1d, Raet1e, Rbm3, Rbx1, Reg3b, Reln, Rnasel, S100a8, S100a9, Saa1, Saa2, Saa3, Saa4, Scara5, Scd1, Serpina3n, Serpine1, Serpine2, Serpinh1, Sfpq, Smarca5, Smarcb1, Socs3, Srprb, Stat3, Tfap2a, Tfrc, Themis2, Trat1, Tyrobp, Ubc, Ucp2, Vcam1, Vsig4, Zbp1	6.3248×10^{-15}
GO:0009611	Response to wounding	45	A2m, Aim2, Apcs, Cd163, Cd44, Chi3l3, Col3a1, Csf1, Cxcl1, Cxcl13, Cyba, Cybb, Fabp5, Fmod, Fn1, Fpr2, Gsn, Hck, Hhex, Hp, Itgal, Itgb2, Itih4, Lbp, Nfkbiz, Nrep, Orm1, Orm2, Pdgfrb, Prok2, Reg3b, Reln, S100a8, S100a9, Saa1, Saa2, Saa3, Saa4, Serpina3n, Serpine1, Serpine2, Stat3, Tfrc, Themis2, Vcam1	1.0954×10^{-12}
GO:0006953	Acute-phase response	17	A2m, Apcs, Cd163, Fn1, Hp, Itih4, Lbp, Orm1, Orm2, Reg3b, Saa1, Saa2, Saa3, Saa4, Serpina3n, Stat3, Tfrc	2.0155×10^{-16}

GO:0008152	Metabolic process	243	4930444A02Rik, A2m, Acpp, Acsl3, Acsm2, Adamdec1, Adamts2, Adamts9, Adck3, Afmid, Agxt2l1, Akr1c14, Aldh18a1, Alox5ap, Ang2, Apcs, Arid5b, Art3, Asns, B3galt1, Bcl6, Bgn, Bub1, C1qa, C1qb, C1qc, Car1, Car13, Car3, Ccnt2, Cd44, Cers6, Ces1d, Ces1e, Ces1f, Cfh, Chi3l3, Cish, Col14a1, Col3a1, Cp, Cpd, Cryl1, Csad, Ctsc, Ctsj, Ctss, Cyb561, Cyba, Cybb, Cyp26a1, Cyp2a5, Cyp2b9, Cyp2c40, Cyp2c55, Cyp2c68, Cyp2j13, Cyp3a13, Dbp, Derl1, Dgkb, Dnajb9, Dnajc10, Dynll1, Efemp1, Ehf, Eif1a, Eif2ak1, Eif2ak3, Fabp2, Fabp5, Far1, Fblim1, Fmod, Fn1, Foxa1, Foxa2, Fpr2, Gadd45a, Gadd45g, Galnt2, Gbp3, Gck, Gfpt1, Ghr, Gm10639, Gmppb, Gnat1, Gpc6, Gsta1, Gsta2, Gsta3, Gsta4, Gstm6, Gstt3, Hand2, Hck, Hectd2, Herc6, Hes6, Hhex, Hp, Hs6st2, Hsd3b2, Hsd3b3, Hsd3b5, Hsd3b6, Hspa1a, Hspa1b, Htra1, Ifi205, Igfbp1, Igfbp3, Il33, Inmt, Ip6k1, Isyna1, Itgb8, Itih3, Itih4, Kif5a, Klk1b26, Klk4, Ldhd, Lepr, Lgals3, Lgmn, Lhx2, Lhx6, Lipc, Lipm, Lipo1, Litaf, Loxl1, Lpl, Lrat, Lrp1, Lrp2, Lum, Ly6e, Lyzl6, Magi3, March1, Mcm5, Me2, Mesdc2, Mettl13, Mme, Mmp12, Mmp16, Mnda, Mtx2, Mybl1, Ncf4, Nfkbiz, Nnmt, Nova1, Nudt10, Nudt11, Ogn, Onecut1, Ostc, Papss2, Pax4, Pax8, Pcgf6, Pdgfrb, Phlda1, Pmaip1, Pou2f2, Prelp, Pros1, Prss23, Prss3, Prtn3, Pten, Ptgds, Ptgs1, Ptpn1, Ptpn2, Ptprc, Ptprg, Qk, Qsox1, Rab30, Rbbp4, Rbm3, Rbx1, Reln, Rgs19, Rhobtb3, Rnasel, Rora, Rpl39l, Rps10, Rrbp1, Runx1t1, S100a8, S100a9, Saa1, Scd1, Sdr9c7, Sec61g, Serpina12, Serpina6, Serpine1, Serpinh1, Sfpq, Shmt1, Slc2a2, Slc3a1, Slc7a8, Smarca5, Smarcb1, Smek2, Snapc2, Soat1, Socs2, Socs3, Sqle, Srprb, Srrm1, Stat3, Steap4, Stk31, Stt3a, Tbx20, Tcf21, Tfap2a, Tfrc, Tgm1, Tmprss11d, Tsc22d1, Tspan8, Ttn, Tuba8, Tyrobp, Ubc, Ucp2, Ugp2, Upp2, Vsig4, Zic3	2.3996×10^{-6}
GO:0006805	Xenobiotic metabolic process	10	Cyp26a1, Gsta1, Gsta2, Gsta3, Gsta4, Hsd3b2, Nnmt, Papss2, Ptgs1, Ugp2	3.7531×10^{-4}
GO:0006629	Lipid metabolic process	42	Acsl3, Acsm2, Akr1c14, Alox5ap, B3galt1, Cers6, Ces1d, Ces1f, Cryl1, Cyp26a1, Cyp2c55, Fabp2, Fabp5, Far1, Ghr, Gpc6, Hsd3b2, Hsd3b3, Hsd3b5, Hsd3b6, Ip6k1, Isyna1, Itgb8, Lepr, Lgmn, Lipc, Lipm, Lipo1, Lpl, Lrat, Lrp1, Lrp2, Pdgfrb, Pten, Ptgds, Ptgs1, Qk, Saa1, Scd1, Serpina6, Soat1, Sqle	4.7674×10^{-4}
GO:0008203	Cholesterol metabolic process	6	Lepr, Lipc, Lrp1, Saa1, Soat1, Sqle	0.03225
GO:0006631	Fatty acid metabolic process	15	Acsl3, Acsm2, Alox5ap, Ces1d, Ces1f, Cryl1, Fabp2, Far1, Ghr, Lipc, Lpl, Ptgds, Ptgs1, Qk, Scd1	0.00203

GO:0042981	Regulation of apoptotic process	46	Asns, Bcl6, Bmp5, Cd44, Cxcl13, Dnajb9, Dynll1, Eif2ak3, Fcer1g, Fn1, Gabra5, Gas1, Hand2, Hck, Hspa1a, Hspa1b, Igfbp3, Inhbb, Lcn2, Lgmn, Lifr, Lrp1, Mnda, Mt1, Pax4, Pax8, Pdgfrb, Phlda1, Plac8, Pmaip1, Prok2, Pten, Ptgfr, Ptprc, S100a8, S100a9, Serpine1, Six4, Slc46a2, Socs2, Socs3, Tfap2a, Timp3, Tsc22d1, Ubc, Ucp2	0.00189
GO:0019221	Cytokine-mediated signaling pathway	23	Aim2, Cd44, Cish, Csf2rb, Csf2rb2, Cxcl13, Ghr, Hck, Icam1, Ifitm2, Il13ra1, Il2rg, Lepr, Lifr, Lilrb3, Ptpn1, Ptpn2, Rnasel, Socs3, Stat3, Ubc, Vcam1, Xaf1	2.6155×10^{-7}
GO:0060397	JAK-STAT cascade involved in growth hormone signaling pathway	7	Cish, Ghr, Prl, Ptpn1, Socs2, Socs3, Stat3	2.275×10^{-7}
GO:0007229	Integrin-mediated signaling pathway	12	Adamts2, Adamts9, Col3a1, Fcer1g, Hck, Itga4, Itga8, Itgal, Itgb2, Itgb8, Nedd9, Tyrobp	4.6443×10^{-6}
GO:0060333	Interferon-gamma-mediated signaling pathway	7	Cd44, Hck, Icam1, Ptpn1, Ptpn2, Socs3, Vcam1	3.525×10^{-5}

The functional properties of DEGs were analysed using the GeneXplain platform and the *p*-value threshold was set as < 0.05.

Supplementary Table S5: Enriched biological processes after repeated treatment for 3 days

GO ID	Biological Process	No of genes	Genes	*P*-value
GO:0006954	Inflammatory response	37	Adora2a, Apcs, B4galt1, Ccr1, Chi3l3, Chi3l4, Chst4, Cxcl1, Cxcl13, Cxcl2, Fcgr3, Hp, Il1rn, Itih4, Lbp, Myd88, Nfkbiz, Orm1, Orm2, Pla2g4c, Pla2g7, Plscr1, Rps6ka5, S100a8, S100a9, Saa1, Saa2, Saa3, Saa4, Serpina3n, Smad1, Stat3, Tfrc, Tirap, Tlr2, Tnfrsf1a, Vcam1	1.4121×10^{-11}
GO:0006955	Immune response	44	Adcy9, Apoa4, Bcl3, Ccl6, Ccr1, Cd55, Chst4, Ctsc, Cxcl1, Cxcl13, Cxcl2, Egfr, Fcgr3, Fgf1, Foxo3, Ifitm2, Il1r1, Il1r2, Il1rn, Irak4, Irgm1, Irs1, Lax1, Lbp, Lcn2, Map3k1, Mapkapk3, Marco, Myd88, Nrg4, Orm1, Plscr1, Pou2af1, Prg4, Rps6ka5, S100a8, S100a9, Saa1, St6gal1, Tirap, Tlr2, Trib3, Ubc, Xbp1	2.9585×10^{-5}
GO:0006950	Response to stress	130	Aacs, Acot2, Adcy9, Adora2a, Adora2b, Ahr, Alas1, Ang, Apcs, Apoa4, B4galt1, Bcl3, Btg1, Cacna1b, Ccnd1, Ccr1, Cd55, Cdkn1a, Chi3l3, Chi3l4, Chl1, Chst4, Clec2h, Creb3l2, Cry1, Cxcl1, Cxcl13, Cxcl2, Cyp17a1, Dhcr24, Dnaja3, Drd1a, Dusp1, Ect2, Egfr, Egr1, Eme2, F13a1, Fabp5, Fcgr3, Fen1, Fgf1, Fgfr1op2, Fnip1, Fnip2, Foxa3, Foxo3, Grm7, Gstm3, Herpud1, Hmgcs2, Hp, Hspa1a, Hspb8, Icam1, Id2, Id3, Ifi47, Ifitm2, Igfbp2, Il1r1, Il1rn, Immt, Irak4, Irgm1, Irgm2, Irs1, Itih4, Lbp, Lcn2, Leap2, Lepr, Map3k1, Mapk8ip3, Mapkapk3, Marco, Myd88, Nedd4l, Nek6, Nfkbiz, Nrep, Nrg4, Nrp1, Orm1, Orm2, Pax6, Pcsk9, Pla2g4c, Pla2g7, Plac8, Plk3, Plscr1, Pnliprp2, Ppargc1a, Ppargc1b, Psmd2, Ptpn1, Ret, Rps6ka5, S100a8, S100a9, Saa1, Saa2, Saa3, Saa4, Scara5, Sell, Serpina3n, Sgk2, Smad1, Socs3, Srd5a1, Srebf2, Stat3, Tardbp, Tbx3, Tff3, Tfrc, Tirap, Tlk1, Tlr2, Tnfrsf1a, Trib3, Tsc22d3, Ubc, Ung, Vcam1, Vcan, Xbp1, Xiap	4.1282×10^{-13}
GO:0009611	Response to wounding	49	Adora2a, Apcs, Apoa4, B4galt1, Ccr1, Chi3l3, Chi3l4, Chl1, Chst4, Cxcl1, Cxcl13, Cxcl2, F13a1, Fabp5, Fcgr3, Fgfr1op2, Hp, Id3, Il1rn, Itih4, Lbp, Map3k1, Myd88, Nfkbiz, Nrep, Nrp1, Orm1, Orm2, Pax6, Pla2g4c, Pla2g7, Plscr1, Rps6ka5, S100a8, S100a9, Saa1, Saa2, Saa3, Saa4, Serpina3n, Smad1, Stat3, Tff3, Tfrc, Tirap, Tlr2, Tnfrsf1a, Vcam1, Vcan	8.4639×10^{-13}
GO:0006953	Acute-phase response	15	Apcs, Hp, Il1rn, Itih4, Lbp, Orm1, Orm2, Plscr1, Saa1, Saa2, Saa3, Saa4, Serpina3n, Stat3, Tfrc	7.7878×10^{-13}

GO:0008152	Metabolic process	291	4930444A02Rik, Aacs, Acaa1a, Acaa1b, Acnat2, Acot1, Acot2, Acpp, Acsl1, Adck3, Adcy9, Adora2a, Agbl4, Agpat6, Agxt2l1, Ahr, Alas1, Aldh18a1, Aldh1b1, Ang, Apcs, Apoa4, Arid5b, Arsj, Atat1, Atp1a2, B3galt1, B3galt2, B3gnt5, B4galnt4, B4galt1, Bcl3, Btg1, Car1, Car13, Car8, Ccnd1, Cd55, Cdc20, Cdkn1a, Cers6, Ces1d, Ces1e, Chi3l3, Chi3l4, Chst4, Chsy3, Clgn, Cml2, Creb3l2, Cry1, Ctbs, Ctsc, Ctsj, Cux2, Cyb561, Cyp17a1, Cyp26a1, Cyp2c39, Cyp39a1, Cyp3a13, Cyp3a44, Cyp4a10, Cyp4a31, Cyp51, Cyp7a1, Dct, Ddc, Decr1, Dhcr24, Dhcr7, Dnaja3, Dner, Dnm3, Dnmt3a, Dpys, Drd1a, Dusp1, Egfr, Egr1, Eif1a, Ell, Elovl2, Eme2, Epha5, Etv6, Eva1a, F13a1, Fabp2, Fabp4, Fabp5, Fbxo21, Fdps, Fen1, Fnip2, Foxa3, Foxo3, G6pc, Gan, Gnat1, Gnpda1, Grpel2, Gstm3, Gstt3, Gzmd, Gzmf, Heatr1, Herpud1, Hes6, Hivep3, Hmgcs1, Hmgcs2, Hnrpdl, Hp, Hs6st2, Hsd17b6, Hsd17b7, Hsd3b5, Hspa1a, Id3, Idi1, Igfbp2, Igtp, Il1rn, Il33, Inmt, Irak4, Irf2bp2, Irgm1, Irgm2, Irs1, Isyna1, Itih3, Itih4, Klf11, Klf17, Lepr, Lhx1, Lin28a, Litaf, Lnx1, Lpgat1, Lpin1, Lpin2, Ly6e, Mafb, Maff, Mafg, Map3k1, Mapk8ip3, Mapkapk3, March7, Mast3, Mat2a, Mcm10, Meis1, Mme, Mmp16, Mucl1, Mvd, Myd88, Nars2, Nedd4l, Nek6, Nfkbiz, Nkx2-2, Nnmt, Nop58, Nova1, Npc1, Nqo2, Nr1i2, Nt5c3, Ocln, Pabpc1, Pak3, Pax6, Pax9, Pck1, Pcsk9, Pdpr, Peg3, Per2, Phb2, Phlda1, Pknox1, Pla2g4c, Pla2g7, Plk3, Plrg1, Plscr1, Pnliprp2, Pot1a, Pou2af1, Ppargc1a, Ppargc1b, Ppp1r3b, Prdm5, Prkag2, Prlr, Prox1, Prtn3, Psmd2, Pspc1, Psph, Ptk6, Ptp4a1, Ptpn1, Ptprd, Qpct, Qsox1, Rab40b, Rbp1, Ret, Retsat, Rnf152, Rpl22, Rpl39l, Rps6ka5, Rrp1b, S100a8, S100a9, Saa1, Sars2, Sc4mol, Scml4, Scyl3, Sgk2, Skor1, Skp2, Slc10a2, Slc23a1, Slc30a5, Slc3a1, Slc7a2, Smad1, Socs3, Sox3, Sox5, Spam1, Spsb4, Sqle, Srd5a1, Srebf2, Srsf12, St3gal1, St3gal4, St3gal5, St6gal1, Stat3, Stat5a, Steap4, Stk31, Sult1e1, Tardbp, Tbr1, Tbx3, Tef, Tfrc, Tgm1, Tk1, Tle3, Tlk1, Tlr2, Tmprss11g, Tmprss2, Tnfrsf1a, Tor3a, Trak2, Treh, Trib3, Tsc22d1, Tspan8, Ttl, Ttll7, Tubb2b, Tubb6, Ubc, Ung, Upp2, Usp2, Usp36, Vcan, Xbp1, Xiap, Zbtb16, Zcchc11, Zfp143, Zfp91, Zic1, Znrd1	2.169×10^{-9}
GO:0006805	Xenobiotic metabolic process	11	Acsl1, Ahr, Cyp17a1, Cyp26a1, Cyp39a1, Cyp7a1, Gstm3, Mat2a, Nnmt, Nr1i2, Sult1e1	2.8224×10^{-4}

GO:0006629	Lipid metabolic process	73	Aacs, Acaa1a, Acaa1b, Acnat2, Acot1, Acot2, Acsl1, Agpat6, Alas1, Ang, Apoa4, Arsj, B3galt1, B3gnt5, Cers6, Ces1d, Cyp17a1, Cyp26a1, Cyp39a1, Cyp4a10, Cyp51, Cyp7a1, Decr1, Dhcr24, Dhcr7, Drd1a, Egfr, Elovl2, Fabp2, Fabp4, Fabp5, Fdps, G6pc, Hmgcs1, Hmgcs2, Hsd17b6, Hsd17b7, Hsd3b5, Idi1, Il1rn, Irs1, Isyna1, Lepr, Lpgat1, Lpin1, Lpin2, Mvd, Npc1, Nr1i2, Pck1, Pcsk9, Pla2g4c, Pla2g7, Plscr1, Pnliprp2, Ppargc1a, Ppargc1b, Prkag2, Prlr, Rbp1, Retsat, Saa1, Sc4mol, Slc10a2, Sqle, Srd5a1, Srebf2, St3gal5, Stat5a, Sult1e1, Tlr2, Tnfrsf1a, Trib3	7.779×10^{-14}
GO:0008203	Cholesterol metabolic process	18	Apoa4, Cyp39a1, Cyp51, Cyp7a1, Dhcr24, Dhcr7, Fdps, Hmgcs1, Hmgcs2, Hsd17b7, Idi1, Lepr, Mvd, Npc1, Pcsk9, Saa1, Sqle, Srebf2	2.3867×10^{-10}
GO:0006631	Fatty acid metabolic process	23	Aacs, Acaa1a, Acaa1b, Acnat2, Acot1, Acot2, Acsl1, Agpat6, Ces1d, Cyp4a10, Decr1, Elovl2, Fabp2, Fabp4, Lpin1, Lpin2, Pla2g4c, Ppargc1a, Prkag2, Sc4mol, Stat5a, Tlr2, Tnfrsf1a	1.551×10^{-6}
GO:0008219	Cell death and apoptosis	47	1810011O10Rik, Adck3, Adora2a, Ahr, Ang, Bmf, Ccl6, Clca2, Cxcl2, Dhcr24, Dnaja3, Ect2, Eva1a, Fgd4, Foxo3, Gan, Gas1, Gzmd, Gzmf, Hspb8, Klf11, Lcn2, Litaf, Nek6, Ocln, Pcsk9, Peg3, Phlda1, Plekhf1, Plk3, Plscr1, Ppargc1a, Psmd2, Rassf5, Rnf152, S100a8, S100a9, Slit2, Sos2, Tardbp, Tnfrsf12a, Tnfrsf1a, Trib3, Ubc, Xiap, Zbtb16, Zc3h12a	9.7358×10^{-5}
GO:0019221	Cytokine-mediated signaling pathway	26	Acsl1, Ccr1, Csf2rb, Csf2rb2, Cxcl13, Egr1, Icam1, Ifitm2, Il13ra1, Il1r1, Irak4, Lepr, Lifr, Myd88, Osmr, Prlr, Ptpn1, Rps6ka5, Socs3, Stat3, Stat5a, Tirap, Tnfrsf1a, Ubc, Vcam1, Zcchc11	6.1013×10^{-8}
GO:0060397	JAK-STAT cascade involved in growth hormone signaling pathway	6	Irs1, Prlr, Ptpn1, Socs3, Stat3, Stat5a	1.0702×10^{-5}
GO:0097190	Apoptotic signaling pathway	14	Bcl3, Bmf, Cdkn1a, Clca2, Ect2, Fgd4, Fnip2, Foxo3, Plscr1, Sos2, Tnfrsf1a, Trib3, Ubc, Xiap	0.01141

The functional properties of DEGs were analysed using the GeneXplain platform and the *p*-value threshold was set as < 0.05.

Supplementary Table S6: Enriched biological processes after repeated diclofenac treatment for 14 days

GO ID	Biological process	No. of hits	Genes	*p*-value
GO:0006954	Inflammatory response	50	A2m, Aoc3, Apcs, Ass1, B4galt1, Bmp6, C4b, Camk1d, Ccr5, Cd14, Cd163, Cd44, Chi3l3, Csf1r, Cxcl1, Cxcl13, Cxcl2, Cyba, Ddt, Ecm1, Fcgr3, Fn1, Gja1, Hmgb1, Hp, Il1rn, Itih4, Lbp, Ly75, Mecom, Mgll, Ncf1, Nfkbiz, Orm1, Orm2, P2rx7, Reg3b, Reg3g, S100a9, Saa1, Saa2, Saa3, Saa4, Sele, Serpina3n, Serpinf2, Stat3, Tfrc, Tlr13, Tnfrsf1a	2.73×10^{-18}
GO:0006955	Immune response	62	Apoa4, Bcl3, Bmp6, C1qb, C2, C4b, C4bp, Ccl6, Ccl9, Ccr5, Cd14, Cd28, Clec4n, Cr2, Csf1r, Ctss, Cxcl1, Cxcl13, Cxcl14, Cxcl2, Cyba, Egfr, Enpp2, Fas, Fcgr3, Fgf3, Fyb, Hamp, Hbegf, Herc6, Hmgb1, Ifitm2, Ifitm3, Iigp1, Ikbke, Il18bp, Il1r1, Il1rn, Jun, Lbp, Lcn2, Ly75, March1, Marco, Mbl1, Ncf1, Ncf4, Nfil3, Orm1, P2rx7, Pik3r1, Prg4, Pros1, S100a9, Saa1, Sarm1, St6gal1, Tlr13, Tnfaip8l2, Vsig4, Xbp1	2.85×10^{-10}
GO:0006950	Response to stress	172	A2m, Acot2, Adrb2, Aldh3a2, Ang, Aoc3, Apcs, Apoa4, Ass1, B4galt1, Bcl3, Bdh1, Bmp6, Brca1, Btg3, C1qb, C2, C230052I12Rik, C4b, C4bp, Camk1d, Camp, Ccnd1, Ccr5, Cd14, Cd163, Cd28, Cd44, Cd9, Cdc42ep5, Cdca5, Cdkn1a, Cgref1, Chac1, Chi3l3, Clec4n, Cpb2, Cr2, Creb3l2, Csf1r, Ctss, Cul7, Cxcl1, Cxcl13, Cxcl2, Cyba, Cycs, Dap, Ddit4, Ddt, Derl3, Dnajb9, Dnajc10, Dnajc3, Ecm1, Edem1, Egfr, Egr1, Ero1l, Eya4, Fabp5, Fads1, Fance, Fas, Fcgr3, Fgb, Fgf3, Fmod, Fn1, Fosl2, Gata6, Gck, Gja1, Gja3, Gsn, Gsta4, Gstm1, Gstm3, Hamp, Hamp2, Hbegf, Herc6, Hmgb1, Hp, Hspa1a, Hspa1b, Hspa8, Hspb1, Icam1, Id2, Idh2, Ier3, Ifi47, Ifitm2, Ifitm3, Ifna9, Igfbp2, Iigp1, Ikbke, Il18bp, Il1r1, Il1rn, Immt, Itih4, Jun, Lbp, Lcn2, Lepr, Ly75, Lyz2, Marco, Mastl, Mbl1, Mecom, Mgll, Mtss1, Ncf1, Nfkbiz, Ngp, Nrep, Opa1, Orm1, Orm2, P2rx7, Pdia6, Pdk4, Pik3r1, Pklr, Plac8, Pld1, Plk3, Pou2f3, Ppargc1b, Ppbp, Pros1, Psmd9, Ptpn1, Rbbp8, Rbm3, Reg3b, Reg3g, S100a9, Saa1, Saa2, Saa3, Saa4, Sarm1, Scd1, Sele, Serpina3n, Serpine1, Serpinf2, Shprh, Slc29a1, Snca, Socs3, Srd5a1, Stat3, Tfrc, Timp1, Tlr13, Tnfaip8l2, Tnfrsf1a, Trp53inp1, Tsc22d2, Ulk2, Usp28, Vldlr, Vsig4, Wipi1, Xbp1	4.19×10^{-24}
GO:0006953	Acute-phase response	21	A2m, Apcs, Ass1, Cd163, Fn1, Hp, Il1rn, Itih4, Lbp, Orm1, Orm2, Reg3b, Reg3g, Saa1, Saa2, Saa3, Saa4, Serpina3n, Serpinf2, Stat3, Tfrc	7.78×10^{-20}

GO:0006629	Lipid metabolic process	90	Abcd2, Acaa1a, Acnat2, Acot1, Acot12, Acot2, Acot4, Acsl1, Afp, Agpat6, Aldh3a2, Alox5ap, Ang, Apoa4, Atp5a1, Atp8b1, B3galt1, B4galt4, Bdh1, Brca1, Cacna1h, Cers6, Ces1d, Ces1f, Ces2a, Chkb, Cpt2, Cryl1, Csf1r, Cyp39a1, Cyp4v3, Cyp7a1, Cyp8b1, Eci1, Egfr, Ehhadh, Elovl2, Elovl5, Enpp2, Fabp2, Fabp4, Fabp5, Fads1, Fads2, G0s2, G6pc, Gata6, Ghr, Gpc4, Gpd2, Hadha, Hsd17b6, Hsd3b5, Il1rn, Isyna1, Ivd, Lepr, Lpgat1, Lpin2, Me1, Mgll, Mtmr7, Nans, Ncf1, P2rx7, Pex11a, Piga, Pik3c2a, Pik3r1, Pik3r5, Plch1, Plcxd2, Pld1, Pnpla2, Ppargc1b, Ptgr1, Rbp1, Retsat, Saa1, Scd1, Sdc4, Smpd3, Snca, Sqle, Srd5a1, St8sia4, Sult1e1, Timp1, Tnfrsf1a, Vldlr	5.47×10^{-19}
GO:0034097	Response to cytokine stimulus	53	1100001G20Rik, Acsl1, Ass1, Camp, Ccr5, Cd14, Cd44, Cish, Csf1r, Cxcl13, Cxcl2, Cyba, Egr1, Fas, Fgb, Fn1, Fosl2, Gapdh, Ghr, Hmgb1, Icam1, Ifi205, Ifitm2, Ifitm3, Iigp1, Il13ra1, Il18bp, Il1r1, Il1rn, Irak3, Irf2, Itih4, Jun, Lcn2, Lepr, Mcl1, Mme, Nfil3, Pid1, Ptpn1, Ptpn2, Sec61a1, Sele, Serpina3m, Serpina3n, Serpine1, Snca, Socs3, Stat3, Tgtp1, Timp1, Tnfrsf1a, Vldlr	3.23×10^{-13}
GO:0055114	Oxidation-reduction process	63	Abcd2, Acaa1a, Aldh1l1, Aldh3a2, Alox5ap, Aoc3, Atp5a1, Bdh1, Cp, Cpt2, Cryl1, Cyb561, Cyba, Cycs, Cyp2c44, Cyp2c70, Cyp39a1, Cyp4a12a, Cyp4v3, Cyp7a1, Cyp8b1, Dnajc10, Eci1, Ehhadh, Ero1l, Fads1, Fads2, G6pc, Gapdh, Gck, Gpd2, Gyg, Hadha, Hsd17b6, Hsd3b5, Hsdl2, Idh2, Idh3b, Ivd, Lepr, Mdh1, Me1, Moxd1, Mthfd1, Ncf1, Ncf4, Ndufa5, Ogdh, Pck2, Pcyox1l, Pdia4, Pdia6, Pid1, Pklr, Ptgr1, Qsox1, Retsat, Scd1, Snca, Sqle, Srd5a1, Steap4, Uba5	1.01×10^{-08}
GO:0034976	Response to endoplasmic reticulum stress	14	Ccnd1, Chac1, Creb3l2, Cul7, Derl3, Dnajb9, Dnajc10, Dnajc3, Edem1, Ero1l, Pdia6, Ptpn1, Wipi1, Xbp1	1.33×10^{-05}
GO:0019221	Cytokine-mediated signaling pathway	23	Acsl1, Ccr5, Cd44, Cish, Csf1r, Cxcl13, Egr1, Fas, Ghr, Icam1, Ifitm2, Ifitm3, Iigp1, Il13ra1, Il1r1, Irak3, Irf2, Lepr, Ptpn1, Ptpn2, Socs3, Stat3, Tnfrsf1a	2.44×10^{-05}
GO:0006006	Glucose metabolic process	14	Eno1, Fabp5, G6pc, Gapdh, Gck, Got1, Gpd2, Hk2, Mdh1, Ogdh, Pck2, Pdk4, Pik3r1, Pklr	2.96×10^{-04}
GO:0008219	Cell death	49	Aco2, Ang, Bcl2l13, Bik, Brca1, C1d, Ccl6, Ccr5, Cd14, Chac1, Cul7, Cxcl2, Cycs, Dap, Dapl1, Ddit4, Dynll1, Ero1l, Fas, Fgd2, Fosl2, G0s2, Gan, Gapdh, Gas1, Gata6, Gja1, Gsn, Hmgb1, Hspb1, Ier3, Irak3, Lcn2, Lyz2, Mageh1, Mcl1, Mecom, Ncf1, Opa1, P2rx7, Plk3, Psmd9, S100a9, Slc9a3r1, Tgm6, Tnfrsf1a, Trp53inp1, Zbtb16, Zc3h12a	5.70×10^{-04}

GO:0006805	Xenobiotic metabolic process	11	Acsl1, Cyp39a1, Cyp7a1, Cyp8b1, Gsta2, Gsta4, Gstm1, Gstm3, Papss2, Sult1e1, Ugt2b1	8.44×10^{-04}
GO:0097193	Intrinsic apoptotic signaling pathway	12	Bcl3, Brca1, Cdkn1a, Chac1, Cul3, Cycs, Ddit4, Dnajc10, Dynll1, Ero1l, Ikbke, Usp28	9.55×10^{-04}
GO:0097190	Apoptotic signaling pathway	18	Bcl3, Brca1, Cd28, Cdkn1a, Chac1, Cul3, Cycs, Dap, Ddit4, Dnajc10, Dynll1, Ero1l, Fas, Fgd2, Igf1, Ikbke, Tnfrsf1a, Usp28	0.00123
GO:0060333	Interferon-gamma-mediated signaling pathway	6	Cd44, Icam1, Irf2, Ptpn1, Ptpn2, Socs3	0.0013
GO:0007259	JAK-STAT cascade	6	Cish, Ghr, Ptpn1, Socs2, Socs3, Stat3	0.00166
GO:0006749	Glutathione metabolic process	6	Alox5ap, Gsta2, Gsta4, Gstm1, Gstm3, Gstt2	0.00357
GO:0012501	Programmed cell death	39	Bcl2l13, Bik, Brca1, C1d, Ccl6, Cd14, Chac1, Cul7, Cycs, Dap, Dapl1, Ddit4, Dynll1, Ero1l, Fas, Fgd2, G0s2, Gapdh, Gas1, Gata6, Gja1, Gsn, Hmgb1, Ier3, Irak3, Lcn2, Mageh1, Mcl1, Mecom, Ncf1, Opa1, Plk3, Psmd9, S100a9, Slc9a3r1, Tnfrsf1a, Trp53inp1, Zbtb16, Zc3h12a	0.004
GO:0002224	Toll-like receptor signaling pathway	9	Cd14, Ctss, Ikbke, Irak3, Jun, Lbp, Reg3g, Sarm1, Tlr13	0.00864
GO:0001666	Response to hypoxia	17	Acot2, Adrb2, Ang, Ddit4, Egr1, Fas, Fosl2, Gata6, Hp, Icam1, Lepr, Pklr, Slc29a1, Socs3, Tfrc, Tnfrsf1a, Vldlr	0.01062
GO:0006979	Response to oxidative stress	17	Aldh3a2, Apoa4, Btg3, Cycs, Egfr, Fn1, Gja3, Hp, Il18bp, Il1r1, Jun, Lcn2, Plk3, Ppargc1b, Serpine1, Snca, Trp53inp1	0.01665
GO:0002764	Immune response-regulating signaling pathway	15	Cd14, Cr2, Ctss, Fcgr3, Fyb, Ikbke, Irak3, Jun, Lbp, Marco, Pik3r1, Reg3g, Sarm1, Tlr13	0.02658

The functional properties of DEGs were analysed using the GeneXplain platform and the *p*-value threshold was set as < 0.05.

Supplementary Table S7: Top bio-functions of differentially expressed genes after single diclofenac treatment

Category	*p*-value	Molecules
**Diseases and Disorders**		
Cancer	3.13E-08-2.92E-02	Pvrl3, Ets2, Sfpq, Serpina3, Tnxb, Nek2, Ptprj, Cxcl13, Apcs, T2a, Lbp, Fcgr3a, Timp3, Arid5b, Orm1/Orm2, Espl1, Il13ra1, Lcn2, Tll1, Slc7a8, Stat3, Hist1h3a (includes others), Mlph, Il18bp, Islr, Prss23, Fam198b, C18orf1, Sik1, Pml, Mt1e, Hmgb2
Inflammatory Response	1.79E-06-2.78E-02	Timp3, Orm1/Orm2, Ear2 (Includes Others), Lcn2, Fpr2, Ets2, Serpina3, Stat3, Il18bp, Cxcl13, Prss23, Ptprj, Prtn3, Apcs, Plcb1, Lbp, Pml, Fcgr3a, Mt1e
Gastrointestinal Disease	1.8E-06-2.78E-02	Pvrl3, Sfpq, Serpina3, Tnxb, Nek2, Ptprj, Cxcl13, Apcs, Mt2a, Cdyl, Fgf23, Plcb1, Lbp, Timp3, Lcn2, Tll1, Stat3, Hist1h3a (Includes others), Mlph, Islr, Prss23, C18orf1, Tcf19, Pml, Mt1e, Hmgb2
Organismal Injury and Abnormalities	9.65E-06-2.48E-02	Mt2a, Lcn2, Fgf23, Stat3, Mt1e
Renal and Urological Disease	9.65E-06-2.78E-02	Timp3, Ptprj, Apcs, Prtn3, Mt2a, Lcn2, Fgf23, Ets2, Serpina3, Pml, Fcgr3a, Mt1e
**Molecular and Cellular Functions**		
Cellular Movement	1.49E-06-2.78E-02	Timp3, Arid5b, Orm1/Orm2, Ear2 (includes others), Lcn2, Fpr2, Gfra2, Serpina3, Stat3, Il18bp, Ptprj, Cxcl13, Prtn3, Apcs, Lbp, Fcgr3a, Hmgb2
Molecular Transport	1.78E-06-2.55E-02	Orm1/Orm2, Fpr2, Gfra2, Stat3, Il18bp, Gnat1, Cxcl13, Ptprj, Mt2a, Fgf23, Sik1, Plcb1, Lbp, Pml, Fcgr3a, Mt1e
Cell Death	9.65E-06-2.89E-02	Timp3, Espl1, Lcn2, Ets2, Serpina3, Gfra2, Stat3, Nek2, Gnat1, Apcs, Prtn3, Mt2a, Lbp, Pml, Mt1e, Fcgr3a, Hmgb2
Cellular Assembly and Organization	9.65E-06-2.79E-02	Timp3, Espl1, Pvrl3, Serpina3, Gfra2, Stat3, Tnxb, Mlph, Nek2, Mt2a, Apcs, Sik1, Mup1(Includes others), Lbp, Pml, Mt1e, Hmgb2
Cellular Compromise	9.65E-06-1.86E-02	Nek2, Prss23, Mt2a, Apcs, Plcb1, Stat3, Pml, Mt1e, Hmgb2
**Physiological System Development and Function**		
Hematological System Development and Function	1.49E-06-3.04E-02	Timp3, Orm1/Orm2, Ear2 (includes others), Lcn2, Fpr2, Ets2, Serpina3, Stat3, Il18bp, Cxcl13, Ptprj, Prtn3, Apcs, Plcb1, Lbp, Pml, Fcgr3a, Hmgb2
Immune Cell Trafficking	1.49E-06-2.78E-02	Timp3, Orm1/Orm2, Ear2 (includes others), Lcn2, Fpr2, Ets2, Serpina3, Stat3, Il18bp, Cxcl13, Ptprj, Apcs, Prtn3, Plcb1, Lbp, Pml, Fcgr3a
Hepatic System Development and Function	9.65E-06-2.49E-02	Mt2a, Lbp, Pml, Meg3, Mt1e
Hematopoiesis	1.44E-04-2.78E-02	Cxcl13, Apcs, Lcn2, Ets2, Plcb1, Stat3, Pml, Hmgb2
Tissue Development	2.33E-04-2.85E-02	Pvrl3, Ets2, Tnxb, Ptprj, Gnat1, Cxcl13, Mt2a, Apcs, Cdyl, Fgf23, Plcb1, Lbp, Arid5b, Timp3, Orm1/Orm2, Lcn2, Fpr2, Tll1, Gfra2, Stat3, Meg3, Il18bp, Prtn3, Pml, Hmgb2

In total, 295 genes were selected based on a cutoff of > 1.5-fold change ($P < 0.05$) using the GenePlex program. Bio-functions were analyzed using the ingenuity pathway analysis program (IPA).

Supplementary Table S8: Top bio-functions of differentially expressed genes after repeated diclofenac treatment for 3 days

Category	*p*-value	Molecules
**Diseases and Disorders**		
Inflammatory Response	4.35E-11-1.15E-02	Socs3, Sell, Sirpb1, Serpina3, Ces1, Cd55/Daf2, Psg18 (includes others), Tnfrsf12a, Ca3, Cxcl3, Wasl, Apcs, S100a8, Lbp, Egfr, Ptpre, Hdac4, Orm1/Orm2, Cd93, Lcn2, Let-7, F13a1, Bcl3, Il1r1, Stat3, Gnaz, Il33, Chi3l3/Chi3l4, S100a9, Prtn3, Ctsc, Cxcl2
Infectious Disease	8.27E-07-1.1E-02	Socs3, Tmprss2, Plk3, Lcn2, Let-7, F13a1, Saa2, Bcl3, Il1r1, Stat3, Cxcl3, Wasl, S100a9, Apcs, Snx10, S100a8, Lbp
Inflammatory Disease	8.27E-07-9.8E-03	Socs3, Sell, Slc1a4, Serpina3, Saa2, Slc39a14, Cd55/Daf2, Cxcl3, Apcs, S100a8, Lbp, Slc41a1, Hdac4, Lcn2, Let-7, F13a1, Il1r1, Stat3, Xiap, Il33, Chi3l3/Chi3l4, S100a9, Prtn3, Cxcl2, Ctsc, Wnt11, Fam214a, Steap4, Ube2i
Respiratory Disease	8.27E-07-1.34E-02	Il33, Chi3l3/Chi3l4, S100a9, Apcs, Lcn2, Let-7, Bcl3, Saa2, Il1r1, Lbp, Stat3, Egfr
Hematological Disease	1.5E-06-1.25E-02	Socs3, Sell, Ptpre, Slc1a4, Hdac4, Slc22a3, Lcn2, Let-7, F13a1, Lrit2, Bcl3, Il1r1, Stat3, Xiap, Il33, Cxcl3, Tacc2, S100a9, Slc37a1, Lbp, Lpin2, Fam214a, Egfr, Ube2i
**Molecular and Cellular Functions**		
Cellular Movement	8.06E-09-1.26E-02	Socs3, Sell, Serpina3, Cd55/Daf2, Tnfrsf12a, Cxcl3, Wasl, Apcs, S100a8, Lbp, Egfr, Il33, Orm1/Orm2, Cd93, Lcn2, F13a1, Stat3, Il1r1, Gnaz, Xiap, Chi3l3/Chi3l4, S100a9, Prtn3, Cxcl2, C5orf13, Wnt11, Ube2i
Cell-To-Cell Signaling and Interaction	6.67E-08-1.26E-02	Socs3, Sell, Apoa4, Sirpb1, Cd55/Daf2, Tnfrsf12a, Cxcl3, Wasl, Apcs, S100a8, Lbp, Egfr, Ptpre, Orm1/Orm2, Hdac4, Cd93, Lcn2, F13a1, Bcl3, Il1r1, Stat3, Il33, S100a9, Prtn3, Cxcl2
Antigen Presentation	1.23E-07-1.1E-02	Socs3, Ptpre, Sell, Lcn2, Cd93, Sirpb1, Serpina3, Stat3, Ces1, Il33, Cxcl3, S100a9, Prtn3, Apcs, S100a8, Lbp, Cxcl2
Lipid Metabolism	3.84E-06-1.22E-02	Apoa4, Saa2, Il1r1, Stat3, Ces1, B3galt1, Acnat1/Acnat2, Lbp, Tnfrsf12a, Il33, Acot2, Gnat1, Apcs, Saa4, Cyp7a1, Acot1
Molecular Transport	3.84E-06-1.13E-02	Sell, Slc1a4, Slc22a3, Apoa4, Saa2, Slc39a14, Ces1, Tnfrsf12a, Cxcl3, Gnat1, Apcs, Saa4, Cyp7a1, S100a8, Wnk4, Lbp, Egfr, Orm1/Orm2, Lcn2, Bcl3, Stat3, Il1r1, Xiap, Il33, S100a9, Acot2, Slc3a1, Cxcl2, Ube2i
**Physiological System Development and Function**		
Hematological System Development and Function	8.06E-09-1.33E-02	Socs3, Sell, Apoa4, Sirpb1, Serpina3, Ces1, Cd55/Daf2, Tnfrsf12a, Cxcl3, Wasl, Apcs, S100a8, Lbp, Egfr, Ptpre, Orm1/Orm2, Lcn2, Cd93, F13a1, Bcl3, Gnaz, Stat3, Il1r1, Xiap, Il33, Chi3l3/Chi3l4, S100a9, Prtn3, Cxcl2, Ube2i, Hdac4
Immune Cell Trafficking	8.06E-09-1.26E-02	Socs3, Sell, Apoa4, Serpina3, Cd55/Daf2, Cxcl3, Wasl, Apcs, S100a8, Lbp, Egfr, Ptpre, Hdac4, Orm1/Orm2, Lcn2, Cd93, F13a1, Bcl3, Gnaz, Il1r1, Stat3, Il33, Cxcl2, Chi3l3/Chi3l4, S100a9, Prtn3
Tissue Development	1.21E-08-1.25E-02	Socs3, Sell, Apoa4, Hdac4, Orm1/Orm2, Lcn2, Bcl3, Il1r1, Stat3, Gnaz, Il33, Cxcl3, S100a9, Apcs, Prtn3, S100a8, Lbp, Cxcl2, Wnt11, Egfr

Tissue Morphology	3.69E-05-1.33E-02	Socs3, Ptpre, Sell, Apoa4, Orm1/Orm2, Lcn2, Stat3, Il1r1, Ces1, Xiap, Tnfrsf12a, Il33, Ca3, Wasl, S100a9, Prtn3, Apcs, S100a8, Cxcl2, Rbbp4, Egfr
Hair and Skin Development and Function	1.76E-04-1.1E-02	Socs3, Sell, Stat3, Egfr

In total, 467 genes were selected based on a cutoff of > 1.5-fold change ($P < 0.05$) using the GenePlex program. Bio-functions were analyzed using the ingenuity pathway analysisprogram (IPA).

Supplementary Table S9: A summary of regulated cytokines and cluster of differentiation molecules (CD) regulated by diclofenac in mice liver after single and repeated treatment

Genes	Day 1 Fold change (average)	Day 3 Fold change (average)	Day 14 Fold change (average)
Il10	−1.04	1.06	−1.05
Il11	−1.00	−1.02	−1.02
Il1b	1.05	1.00	2.60
Il15	−1.19	1.06	1.07
Il17ra	1.48	1.71	2.22
Il18	−1.09	−1.05	1.15
Il2	−1.19	−1.33	1.33
Il20	−1.07	−1.01	1.12
Il21	1.16	1.11	1.05
Il22	−1.08	1.30	1.11
Il24	1.11	1.09	−1.15
Il25	−1.08	−1.13	1.00
Il3	−1.05	1.04	−1.03
Il31	1.24	−1.03	1.26
Il33	1.74	1.54	4.41
Il34	1.01	−1.11	−1.04
Il4	1.09	1.22	1.10
Il5	1.06	1.06	1.05
Il6	1.08	1.15	4.04
Il7	1.20	1.33	1.03
Il9	−1.17	−1.02	−1.08
Irf2	−1.07	−1.07	1.48
Tcra	−1.13	−1.08	−1.05
Tnf	1.08	−1.06	−1.14
Tgfb	−1.23	1.3	−1.28
Cxcl1	17.36	19.1	25.73
Cxcl2	2.3	1.61	11.74
Cxcl13	1.98	2.02	14.62
Cxcl14	2.93	1.06	4.12
Ccl6	2.99	1.68	3.66
Cd4	−1.04	−1.06	1.38
Cd14	1.78	1.48	34.68
Cd163	1.73	1.35	2.51
Cd177	1.72	−1.10	1.71
Cd28	2.31	1.21	5.82

Cd36	1.52	1.16	1.21
Cd44	1.67	1.49	1.83
Cd74	1.42	1.06	1.06
Cd9	1.33	1.31	1.76
Cd8	1.09	−1.34	1.10

Supplementary Table S10: Summary of transcription factor binding site search results for DEGs after single diclofenac treatment. The TRANSFAC® 2013.2 library of positional weight matrices and 'vertebrate_non_redundant_minSUM' profile were used to identify enriched transcription factor binding sites in the promoters of genes coding for inflammation, immune and stress response. Promoters of annotated genes were interrogated for cis-regulatory binding sites of genomic sequences with a length of −1000 to +100 bp relative to TSS. The first ATG codon was considered as tentative TSS (transcription start site). Each significant PWM ($p < 0.05$) is mentioned with Yes density per 1000bp, No density per 1000bp and Yes-No ratio values.

Supplementary Table S11: Transcription factor binding site search results for DEGs after repeated treatment of mice for 3 days

Supplementary Table S12: Transcription factor binding site search results for DEGs after repeated treatment of mice for 14 days

Supplementary Table S13: Number of transcription factor binding sites in promoters of regulated genes after single diclofenac treatment

Supplementary Table S14: Number of transcription factor binding sites in promoters of regulated genes after repeated diclofenac treatment for 3 days

Supplementary Table S15: Number of TFBS in the composite modules of inflammation, immune and stress response DEGs after repeated diclofenac treatment (day 14)

Supplementary Table S16: Common DEGs identified in mouse hepatocyte cultures and *in vivo* findings after single and repeated diclofenac treatment of mice

Genes	Description	*In vitro* (Fold change)	Day 1 (Fold change)	Day 3 (Fold change)	Day 14 (Fold change)
Agxt2l1	Alanine-glyoxylate aminotransferase 2-like 1	−5.66	−1.79	−2.18	−3.16
Cxcl1	Chemokine (C-X-C motif) ligand 1	−13.91	17.36	19.1	25.73
Dnajc12	DnaJ (Hsp40) homolog, subfamily C, member 12	−2.97	3.29	2.70	2.09
Fndc3b	Fibronectin type III domain containing 3B	2.33	2.99	2.04	2.28
Hp	Haptoglobin	−3.80	1.43	1.57	1.44
Hsd3b5	Hydroxy-delta-5-steroid dehydrogenase, 3 beta- and steroid delta-isomerase 5	2.47	−2.42	−1.63	−1.43
Hspa1a	Heat shock protein 1A	2.47	1.71	−1.45	1.68
Icam1	Intercellular adhesion molecule 1	−6.63	1.75	1.85	2.01
Il33	Interleukin 33	−3.51	1.74	1.54	4.41
Lbp	Lipopolysaccharide binding protein	−4.60	1.88	1.67	−1.69
Ly6e	Lymphocyte antigen 6 complex, locus E	−8.25	1.58	1.69	2.40
Marco	Macrophage receptor with collagenous structure	−2.82	4.00	1.98	7.20
Nfkbiz	Nuclear factor of kappa light polypeptide gene enhancer in B-cells inhibitor, zeta	−3.28	2.90	1.69	3.33
Saa4	Serum amyloid A 4	−3.16	1.78	2.18	2.30
Serpina10	Serine (or cysteine) peptidase inhibitor, clade A (alpha-1 antiproteinase, antitrypsin), member 10	−22.74	1.95	1.91	3.05
Slc25a30	Solute carrier family 25, member 30	−7.31	−1.75	−2.10	−1.64
Slco1a4	Solute carrier organic anion transporter family, member 1a4	−3.68	−1.50	−1.43	−1.54
Steap4	STEAP family member 4	−21.91	3.02	4.81	14.58
Tmem176a	Transmembrane protein 176A	−10.91	1.79	1.52	2.21
Tsc22d1	TSC22 domain family, member 1	−10.51	−1.67	−1.55	−1.43
